# Fabrication of Poly(3-hydroxybutyrate-co-3-hydroxyhexanoate)/ZnO Nanocomposite Films for Active Packaging Applications: Impact of ZnO Type on Structure–Property Dynamics

**DOI:** 10.3390/polym16131861

**Published:** 2024-06-29

**Authors:** Chris Vanheusden, Pieter Samyn, Thijs Vackier, Hans Steenackers, Jan D’Haen, Roos Peeters, Mieke Buntinx

**Affiliations:** 1 Materials and Packaging Research & Services, Institute for Materials Research (IMO-IMOMEC), Hasselt University, Wetenschapspark 27, 3590 Diepenbeek, Belgium; chris.vanheusden@uhasselt.be (C.V.); roos.peeters@uhasselt.be (R.P.); 2 Department Circular Economy and Renewable Materials, SIRRIS, Gaston Geenslaan 8, 3001 Leuven, Belgium; pieter.samyn@sirris.be; 3 Department of Microbial and Molecular Systems, Centre of Microbial and Plant Genetics (CMPG), KU Leuven, 3001 Leuven, Belgium; thijs.vackier@kuleuven.be (T.V.); hans.steenackers@kuleuven.be (H.S.); 4 Analytical & Microscopical Services, Institute for Materials Research (IMO-IMOMEC), Hasselt University, Wetenschapspark 1, 3590 Diepenbeek, Belgium; jan.dhaen@uhasselt.be

**Keywords:** polyhydroxyalkanoates, poly(3-hydroxybutyrate-co-3-hydroxyhexanoate), melt processing, extrusion, zinc oxide, nanocomposites, packaging

## Abstract

Bio-based and biodegradable polyhydroxyalkanoates (PHAs) have great potential as sustainable packaging materials. The incorporation of zinc oxide nanoparticles (ZnO NPs) could further improve their functional properties by providing enhanced barrier and antimicrobial properties, although current literature lacks details on how the characteristics of ZnO influence the structure–property relationships in PHA/ZnO nanocomposites. Therefore, commercial ZnO NPs with different morphologies (rod-like, spherical) and silane surface modification are incorporated into poly(3-hydroxybutyrate-co-3-hydroxyhexanoate) (PHBHHx) via extrusion and compression molding. All ZnO NPs are homogeneously distributed in the PHBHHx matrix at 1, 3 and 5 wt.%, but finer dispersion is achieved with modified ZnO. No chemical interactions between ZnO and PHBHHx are observed due to a lack of hydroxyl groups on ZnO. The fabricated nanocomposite films retain the flexible properties of PHBHHx with minimal impact of ZnO NPs on crystallization kinetics and the degree of crystallinity (53 to 56%). The opacity gradually increases with ZnO loading, while remaining translucent up to 5 wt.% ZnO and providing an effective UV barrier. Improved oxygen barrier and antibacterial effects against *S. aureus* are dependent on the intrinsic characteristics of ZnO rather than its morphology. We conclude that PHBHHx retains its favorable processing properties while producing nanocomposite films that are suitable as flexible active packaging materials.

## 1. Introduction

Petroleum-based plastics have become an integral part of modern life because they are lightweight, flexible, cost-effective, and easy to process; however, their environmental impact is undeniable. Plastics such as polypropylene and polyethylene can persist in the environment for centuries, contributing to the pollution of land, rivers, and oceans. The growing global plastic production [1] also contributes to the depletion of fossil fuel reserves, as 4–8% of global oil production is consumed by plastics, with predictions as high as 20% by 2050 [2]. In response to these challenges, bioplastics have emerged as a potential solution because they are produced from alternative feedstocks and can contribute to a circular economy through composting, biodegradation, or other forms of recycling [3,4]. Bioplastic production is predicted to grow continuously over the next few years [5], but its share in total plastic production remains below 1% [6]. The main advantage of bioplastics is that these materials can be processed via conventional conversion technologies like extrusion and injection molding into products with a range of mechanical properties, suitable for packaging [7].

Current drivers of the bioplastic market include innovative polyhydroxyalkanoates (PHAs). PHAs are bio-based and biodegradable polymers produced by bacteria from a range of renewable substrates, such as waste streams [8,9], with broad application potential [10]. Poly(3-hydroxybutyrate) or PHB has proper stiffness but is more brittle with low thermal stability and flexibility [11]. These drawbacks can be overcome by copolymerization with hydroxyvalerate (HV) or hydroxyhexanoate (HHx) units to obtain flexible poly(3-hydroxybutyrate-co-3-hydroxyvalerate) (PHBV) and poly(3-hydroxybutyrate-co-3-hydroxyhexanoate) (PHBHHx). PHBHHx is more ductile compared to PHB and PHBV, with improved processability, lower crystallinity, and lower melting temperature (which is highly dependent on the 3-HHx content) [12,13,14]. However, the functional properties of PHBHHx should be further optimized to broaden its applications, e.g., in food packaging. One possible approach is to incorporate nanoparticles into the polymer matrix.

Inorganic NPs are used in a wide range of applications because they are often stable at high temperatures and some are considered non-toxic to the environment. Metallic NPs such as ZnO, Ag, TiO_2_, and CuO show promise for use in food packaging applications because of their ability to improve the gas barrier and add antibacterial properties [15]. Zinc oxide nanoparticles (ZnO NPs) have attracted significant attention because of their low cost, good availability, UV absorption characteristics, and unique antibacterial properties [16]. ZnO is generally accepted as safe (GRAS) by the FDA [17] and can be used in food contact materials in accordance with the specific migration limit (SML), as regulated by the EFSA [18]. Regarding the end-of-life scenario, ZnO NPs can accelerate the disintegration of bioplastics under hydrolytic or composting conditions, as shown for PLA/ZnO [19,20] or PBAT/PLA/ZnO [21], respectively.

A growing body of literature has investigated PHA/ZnO nanocomposites, but there is no general agreement on the exact influence of ZnO NPs on the processing–structure–property relationships, and specifically on the crystallization behavior of PHAs. Numerous studies found a delay in the crystallization of PHB, PHBV, and PHBHHx with ZnO NPs, by reducing the crystallinity and/or crystallization temperature (T_c_) [22,23,24,25,26,27,28,29,30]. Similarly, a reduction in crystallization temperature for PHB/ZnO with an increase in crystallinity at intermediate ZnO levels was demonstrated [31]. Other studies did not show notable effects of ZnO NPs on the total crystallinity and crystallization rate of PHB and PHBV [32,33], neither accelerating nor inhibiting the crystallization [34]. On the contrary, ZnO can also accelerate the crystallization of PHB, PHBV, and PHBHHx with a rise in crystallization temperature and/or with higher crystallinity [35,36,37,38,39,40]. The reported effects were mostly concentration-dependent, diminishing at elevated ZnO concentrations due to the formation of agglomerates. The mentioned studies reported acceptable dispersion of ZnO without the need for NP surface treatments, despite the fact that improvements in polymer properties are often hindered by poor interfacial compatibility. Only a few studies have been conducted on how surface treatments (e.g., silanization) affect the properties of PHA/ZnO nanocomposites. Bekat and Öner showed that silane treatment of ZnO NPs slightly improved the dispersion quality compared to untreated ZnO NPs, but it reduced the crystallinity of PHBV regardless of the surface treatment [41]. In parallel, the incorporation of ZnO into PHAs can increase or add functional properties, including increased mechanical strength and Young’s modulus together with a reduction in elongation [35], improved oxygen barrier [35], water vapor barrier [35], UV barrier [42], as well as antibacterial effects [35]. As some effects are still dubious, some studies demonstrated improved thermal stability [24,35,43], whereas others also reported reduced thermal stability of PHA/ZnO nanocomposites [22,39].

The further industrialization of polymeric nanocomposites as active food packaging materials requires the applicability of production processes on an industrial scale without the need for toxic solvents. Previous research on the production of PHA/ZnO nanocomposites has mainly focused on solvent-assisted production methods such as solvent casting [27,35,37,42,43,44,45,46], or spinning techniques [22,24,25,36,47]. One approach involves the use of a simple solvent casting technique where PHAs are dissolved in organic solvents (e.g., chloroform) and sonicated in the presence of ZnO NPs. Other methods include a combination of solvent-assisted pre-incorporation followed by further dispersion of the NPs via melt-mixing [28,40]. Only a few studies reported on the fabrication of PHA/ZnO compounds via melt processing [23,31,32,34,39], or a combination of an ultrasonication step prior to melt-processing to improve the dispersion of ZnO in PHBV [26,41].

Present literature fails to explain the large variety in structures and properties of PHA nanocomposites (in particular PHBHHx/ZnO), related to the influences of multiple ZnO NP characteristics such as morphology (shape, size) and surface treatments. Therefore, the objective of this study is to incorporate commercial ZnO NPs with different characteristics into PHBHHx matrices at different concentrations using scalable melt-mixing processes, such as extrusion and compression molding. More specifically, we aim to study the influence of ZnO characteristics such as surface silanization, shape (i.e., rod-like vs. spherical), and size (i.e., diameter 30 to 80 nm and different size distributions) on the structure–property relationship in detail.

## 2. Materials and Methods

### 2.1. Materials

PHBHHx powder (Green Planet^TM^) was kindly supplied by Kaneka Corporation (Westerlo-Oevel, Belgium). Table 1 summarizes the commercial ZnO NP powders used in this study, including different morphologies and/or surface functionalities. Three types of rod-like (R) ZnO NPs (Tenray Z2, Tenray Z2P, and Tenray Z2E) were supplied by EverCare (Eijsden, The Netherlands). The Tenray Z2P and Tenray Z2E ZnO NPs were treated with silane (Si) and silane with ester (Si-e) functionalization, respectively (Table 1). Spherical (S) ZnO NPs (Nanosun P99/30, NM-112) were supplied by Micronisers (Melbourne, Australia), or purchased from Sigma-Aldrich (no. 544906, Merck, Darmstadt, Germany). Ethanol (absolute) was purchased from VWR-Avantor (Leuven, Belgium).

### 2.2. Methods

#### 2.2.1. ZnO NP Characterization

To analyze the ZnO NP morphology and size, transmission electron microscopy (TEM) measurements were performed using a Tecnai Spirit TEM operating at 120 kV (FEI company, Hillsboro, OR, USA). The ZnO NPs were dispersed in ethanol, drop-casted, and dried on a TEM grid (formvar foil upon copper grids, Electron Microscopy Sciences).

To obtain an indication of the difference in hydrophobicity between the different ZnO NP types, contact angle (CA) measurements were performed on dried ZnO films [48]. One mL of ZnO NP dispersions in ethanol (10 mg/mL) were drop-casted and dried on glass standard microscopy slides at 50 °C. The indicative wetting behavior of the ZnO NP layers on glass was tested by water CA measurements (sessile drops), performed with an OCA20 instrument (DataPhysics, Filderstadt, Germany) and SCA 20 software (Version V3.61.4). The D.I. water droplets of 0.5 μL were deposited on the surface using a Hamilton syringe. A droplet of 5 µL was used for the ZnO(R-Si) type because of its very high hydrophobicity. The ZnO surface area, pore volume, and pore size were examined with a TriStar II Plus analyzer (Micromeritics Instruments, Norcross, GA, USA). The ZnO nanopowders were degassed overnight under a nitrogen atmosphere at 150 °C.

#### 2.2.2. PHBHHx/ZnO Film Fabrication

ZnO NPs and PHBHHx powder were oven-dried for at least 3 days at 65 °C and dry-mixed in a Speed Mixer (Synergy Devices Ltd., High Wycombe, UK) at concentrations of 1, 3, and 5 wt.% ZnO. According to the literature, these are commonly used ZnO concentrations [22,49,50,51], while the higher concentrations are less economically feasible and often lead to excessive agglomeration [40]. The higher ZnO concentrations also result in decreased transparency of the material, could possibly increase the migration of ZnO to the packed food and/or could reduce specific functional properties. The dry-mixed ZnO NPs and PHBHHx powder was processed into strands with a twin-screw extruder (Process 11, Thermo-Scientific, Karlsruhe, Germany) equipped with standard screws with a diameter of 11.0 mm and L/D ratio of 40.0. The extrusion temperature profile was set from the feeder to the die as follows: 140-140-150-155-155-160-165-165 °C [13]. The strands were pelletized and dried for a minimum of 24 h at 65 °C and subsequently compression molded in a hydraulic hot press (Henan Chuanghe Laboratory Equipment Co. Ltd., Zhengzhou, China) into films with a size of 10 × 10 cm² and thickness of 180 ± 10 µm. Approximately 2.2 g of pellets were preheated in a stainless-steel mold (150 µm thickness) between Teflon sheets for 4 min at 155 °C without pressure, followed by pressure cycles of 3 and 15 MPa at 155 °C (both for 2 min) and air cooled for 10 min at ambient conditions under light pressure.

#### 2.2.3. Cross-Sectional Film Morphology

The size and distribution of ZnO NPs in the nanocomposite films were analyzed from backscattered electron (BSE) images with inherent Z-contrast captured by scanning electron microscopy (SEM), using a 450 FEGSEM with Gemini 2 optics (ZEISS, Zaventem, Belgium) with accelerating voltage of 10 kV under high vacuum conditions. The cross sections were prepared by cutting under liquid nitrogen conditions and gold-palladium sputtering. An optimized magnification was used as a balance between a resolution of about 50 nm to reveal the ZnO NPs while avoiding electron-beam-induced damage.

#### 2.2.4. Chemical Properties

The chemical composition of the nanocomposite films was analyzed with attenuated total reflectance Fourier transform infrared spectroscopy (ATR-FTIR) by using a Tensor 27 Fourier Transform IR spectrometer equipped with a diamond/ZnSe crystal (Bruker Optics, Kontich, Belgium). The spectral region was analyzed after baseline correction and normalization in the wavelength range between 4000 cm^−1^ and 600 cm^−1^ with a resolution of 4 cm^−1^ and averaged over 16 scans.

#### 2.2.5. Thermal Properties

The melting and crystallization of the nanocomposite films were evaluated using DSC measurements under an inert atmosphere (50 mL/min nitrogen) using a Q200 instrument (TA Instruments, New Castle, DE, USA). Samples of about 6 mg (two distinct film samples per nanocomposite) in sealed aluminum pans were heated from −30 °C to 170 °C, before being kept isothermal for 2 min. Then, the samples were cooled to −30 °C and kept constant for 2 min before heating up to 170 °C. The heating/cooling rate was 20 °C/min.

The degree of crystallinity ($X_{C,DSC}$) was calculated using the following Equation (1):

(1) $$X_{C,DSC}\left(\%\right)=\frac{∆H_{m}}{∆H_{m}^{0}\times \omega_{PHBHHx}}\times 100$$

where Δ*H*_*m*_ is the melting enthalpy from the first heating scan, $∆H_{m}^{0}$ is the melting enthalpy of the 100% crystalline sample (115 J/g) [52,53,54], and *ω*_*PHBHHx*_ is the weight fraction of PHBHHx in the nanocomposites. The crystallization enthalpy $∆H_{c}$ was derived from the first cooling exotherm. The relative degree of crystallinity ($X_{t}$) as a function of crystallization time (t) was calculated for all nanocomposites and is calculated via integration of the first cooling exotherm between the onset and end of crystallization time, according to Equation (2):

(2) $$X_{t}\left(\%\right)=\frac{\int_{0}^{t}\left(\frac{dH}{dt}\right)dt}{\int_{0}^{\infty }\left(\frac{dH}{dt}\right)dt}\times 100$$

The non-isothermal half crystallization time t_1/2_, (crystallization time at 50% relative crystallinity) and the crystallization rate R (1/t_1/2_) were obtained from the relative crystallinity ($X_{t}$) versus crystallization time (t) curves. The exothermic and endothermic enthalpies are reported with respect to the polymer weight fraction. The thermal properties are reported as the average of two measurements.

The thermal stability of the films was analyzed by thermogravimetric analysis (TGA) using a TGA 55 instrument (TA Instruments, New Castle, DE, USA). Samples of approximately 10 mg were weighed in high-temperature ceramic pans and heated from 25 °C to 550 °C at a heating rate of 20 °C/min under a nitrogen gas flow of 90 mL/min.

#### 2.2.6. Crystallographic Properties

The nanocomposite crystal structure was analyzed with wide-angle X-ray diffraction (WAXD) using a D8 Discover apparatus (Bruker Optics, Kontich, Belgium) in the range 2*θ* = 5° to 60° using a Cu source with wavelength λ = 1.54 Å and step size of 0.04°. The degree of crystallinity ($X_{C,XRD}$) was calculated as described in Supplementary Materials S1.

#### 2.2.7. Mechanical Properties

The mechanical properties of the nanocomposites were studied by tensile testing using a benchtop 5ST universal tester (Tinius Olsen, Redhill, UK). Dumbbell-shaped specimens (ASTM D638 type 5, width = 3.18 mm, clamping distance = 25.4 mm and thickness ~180 µm) were cut from the films and were tested at 23 °C and 50% RH with a preload of 0.5 N, a preload speed of 1 mm/min and a crosshead speed of 1 mm/min. The test samples were conditioned for at least 3 days at 23 °C and 50% relative humidity (RH) before tensile testing. The tensile strength (σ) was calculated as the maximum peak stress, the elongation at break ($\varepsilon_{b}$) as the strain value at sample breakage and the Young’s modulus (E) as the slope of linear regression in the strain interval 0.05–0.25%. The mechanical properties are reported as the average and standard deviation of five measurements.

#### 2.2.8. Wetting Properties

Water contact angle experiments (sessile drops) were performed with an OCA20 instrument (DataPhysics, Filderstadt, Germany) and SCA 20 software (Version V3.61.4). Water droplets of 0.5 µL were deposited on the surface of the PHBHHx and PHBHHx/ZnO films. The contact angle at time zero was obtained by extrapolating the linear part of the contact angle versus the time curve by linear regression. The contact angle is reported as the average and standard deviation of five measurements.

#### 2.2.9. Gas Permeability Properties

The oxygen transmission rate (OTR) was measured at 23 °C and 0% RH (ASTM D3985), using an Ox-Tran 702 (Ametek MOCON, Minneapolis, MN, USA). Oxygen gas (99.9990% purity) was purchased from Air Liquide (Brussels, Belgium). Samples were placed between two aluminum masks with an effective testing area of 5 cm^2^ and sealed with epoxy glue. The final test area was measured. The samples were exposed to the test gas (oxygen, humidified in nitrogen) on one side and to a carrier gas (Formier^®^ gas) on the other side, both at a total pressure of 1 atm. The measured OTR was normalized to the thickness of the test samples and expressed as the oxygen permeability coefficient (PO_2_). Samples were measured at least twice and in duplo for each sample type.

#### 2.2.10. UV Barrier Properties

The UV transmittance of the nanocomposite films was determined with a Carry 5000 UV/VIS/NIR spectrophotometer (Agilent Technologies, Santa Clara, CA, USA). The UV/VIS spectra were recorded in the wavelength range of 200–800 nm and expressed as a percentage of transmission. Air was used as the blank reference. The UV/VIS transmission is reported as the average and standard deviation of three measurements.

#### 2.2.11. Optical Properties

Colorimetric and opacity analysis was performed using a Check 3 spectrophotometer (Datacolor, Luzern, Switzerland). The opacity Y (in %) can be calculated as the ratio between the opacity of a sample on a black standard (Y_b_) and the opacity on a white standard (Y_w_). Colorimetric results are presented in CIELAB color coordinates: L*, a*, and b* represent the degree of lightness, and green/red and blue/yellow coordinates, respectively. The total color change, ∆*E*, is calculated via the following Equation (3):

(3) $$∆E=\sqrt{∆L^{2}+∆a^{2}+∆b^{2}}$$

with ∆L, ∆a and ∆b, the difference in L*, a* and b* between the blank PHBHHx (0 wt.% ZnO NPs) and the PHBHHx/ZnO samples. The opacity and colorimetric properties are measured on two distinct samples and reported as the average and standard deviation (SD) of 8 (opacity) and 10 (color) values, respectively.

#### 2.2.12. Antibacterial Activity

*Escherichia coli* TG1 and *Staphylococcus aureus* ATCC 6538 were used for antibacterial testing. Prior to each experiment, the strains were inoculated in Lysogeny broth (LB) and incubated overnight (ONC) at 37 °C (200 rpm). Thereafter, the bacteria were normalized to ~6 × 10^5^ CFU/mL for the minimum inhibitory concentration (MIC) assay and ~1 × 10^7^ CFU/mL to evaluate the antimicrobial activity of the PHBHHx/ZnO films.

The MIC of the different ZnO NP types for both bacteria was tested based on the EUCAST broth microdilution [55], with slight modifications. Briefly, twofold serial dilutions of the different ZnO types in Mueller Hinton Broth (MHB) were prepared in flat-bottom 96-well plates. The plates were inoculated with the normalized ONC resulting in an inoculation of 3 × 10^5^ CFU/mL in a total volume of 200 µL in each well. Moreover, a growth control (MHB with bacteria and without ZnO), a sterile control (MHB without bacteria and ZnO), and a dilution series without bacteria (ZnO control) were included. To avoid evaporation, the plates were sealed using a membrane. These plates were then incubated overnight at 37 °C (200 rpm). The next day, the OD_595_ was measured using a multimode reader BioTek Synergy Mx (Agilent Technologies, Diegem, Belgium). The OD595 was corrected by subtracting the OD595 of the ZnO control or sterile control, whichever was relevant. The MIC was defined as the lowest ZnO concentration at which the corrected OD595 reached the threshold of corrected OD595 > corrected OD595 of the sterile control + 2 SD of the sterile control.

To test the antibacterial activity of the nanocomposite films, pieces of 10 mm × 10 mm of the PHBHHx and PHBHHx/ZnO films were first sterilized in ethanol for 10 min, dried for at least 30 min and placed in separate wells of sterile 12-well plates. Next, 100 µL of the normalized ONC in phosphate-buffered saline (PBS) were pipetted onto the specimen surfaces. The internal volume of the 12-well plates (between the wells) was filled with sterile water to ensure humidity. The specimens were incubated for 24 h at 37 °C. The bacteria were recovered from the film surfaces by washing each sample with 3 × 1 mL of PBS. Each specimen with its washing fluid was transferred to a falcon tube filled with 2 mL of PBS and vortexed for 30 s. Then, tenfold serial dilutions were made in PBS up to 10^−5^, and these dilutions were spot-plated in triplicate on LB agar plates. The plates were incubated overnight at 37 °C. The number of colonies was recorded and used to determine the number of viable bacteria (CFUs/mL) per biological origin. The survival ratio (SR) per biological origin was calculated according to Equation (4):

(4) $$SR\left(\%\right)=\left(\frac{N}{N_{0}}\right)\times 100$$

where N and $N_{0}$ are the average number of colonies (CFUs/mL) on the pure PHBHHx and the nanocomposite films, respectively. The SR is reported as the average of the SR of at least three biological origins per sample type. This value represents at least nine film samples.

#### 2.2.13. Statistical Analysis

The data from tensile testing and contact angle measurements were analyzed to assess statistical differences between the PHBHHx reference films and PHBHH/ZnO nanocomposites. One-way analysis of variance (ANOVA) with Tukey’s multiple mean comparison test was performed using Origin Pro^®^ software (version V9.7.0.188) to compare the data at a significance level of α = 0.05.

## 3. Results and Discussion

### 3.1. Morphological Characteristics of ZnO NPs and PHBHHx/ZnO Nanocomposites

**Figure 1:**
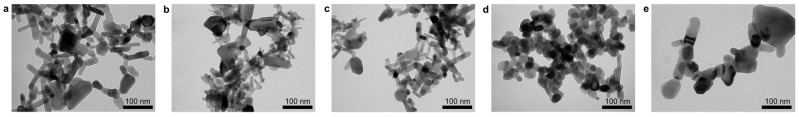
TEM images of ZnO NP types used in this work: (**a**) ZnO(R), (**b**) ZnO(R-Si), (**c**) ZnO(R-Si-e), (**d**) ZnO(S-1), and (**e**) ZnO(S-2).

In order to study the influence of morphology and/or surface functionalization of ZnO NPs on the structure–property relationships of PHBHHx/ZnO nanocomposites, the rod-like and spherical ZnO NPs with different sizes and different surface treatments (Table 1) were first characterized by TEM (Figure 1). The physicochemical properties are shown in Table 2. A more detailed particle size analysis was conducted after image processing of TEM microscopy (see Supplementary Materials, Figure S2). The rod-like ZnO(R), ZnO(R-Si), and ZnO(R-Si-e) appear as rather irregular rods with different aspect ratios L/D and maximum rod lengths up to L = 120 nm. The spherical ZnO(S-1) and ZnO(S-2) have more regular shapes with different diameters D, while the size distribution of ZnO(S-2) is broader. The pore size and volume of pores of rod-like ZnO(R), ZnO(R-Si), ZnO(R-Si-e), and ZnO(S-1) NPs are comparable but strongly differ for ZnO(S-2) in parallel with a strong reduction in surface area. The ZnO(R), ZnO(R-Si-e), ZnO(S-1), and ZnO(S-2) NPs are hydrophilic with indicative contact angles below 20° as determined on a dried film. The silane-modified ZnO(R-Si) has a contact angle above 160° on dried films, demonstrating its hydrophobic properties [48]. The hydrophilic nature of ZnO(R-Si-e) indicates a rather low content of MPS on the surface of the NPs, estimated at around ~1%, compared to ~4% TEOS for ZnO(R-Si) [50].

**Figure 2:**
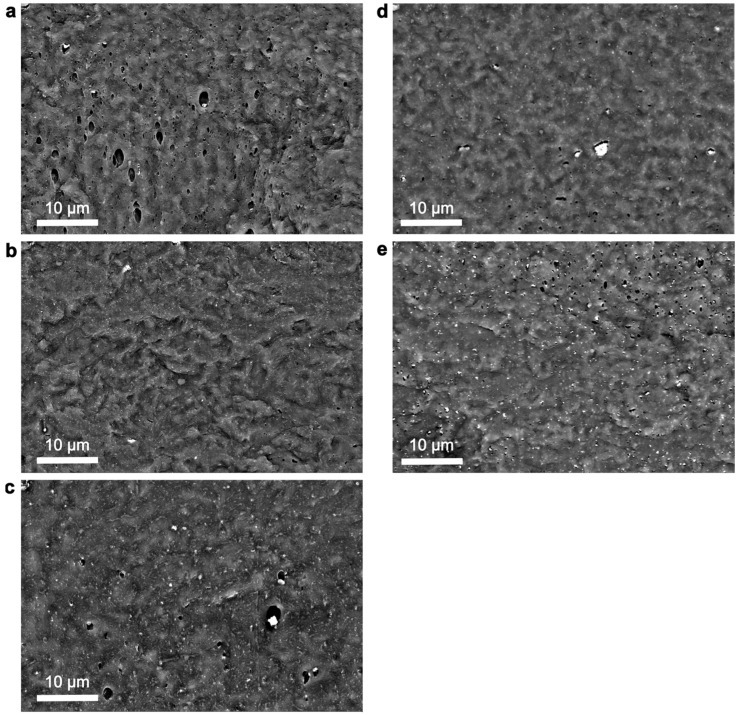
SEM cross-sectional images for 5 wt.% PHBHHx/ZnO nanocomposites with different ZnO NP types: (**a**) ZnO(R), (**b**) ZnO(R-Si), (**c**) ZnO(R-Si-e), (**d**) ZnO(S-1), and (**e**) ZnO(S-2).

The morphology and dispersion state of ZnO NPs in the PHBHHx matrix play crucial roles in the final material properties. Cross-sectional SEM images of 5 wt.% loaded PHBHHx/ZnO films are shown in Figure 2, as the highest concentration should represent the most difficult dispersion state. The size distributions of ZnO NPs in the SEM images were calculated (Supplementary Materials, Figure S3) to provide a better indication of the aggregation and dispersion state of the nanocomposites.

On a macro-to-micro scale, all ZnO types are homogeneously distributed throughout the PHBHHx matrix with only a low number of aggregates. The average and maximum ZnO NP diameters (Feret’s), respectively, are 133 nm, 1.2 µm (ZnO(R)); 119 nm, 1.5 µm (ZnO(R-Si)); 175 nm, 1.7 µm (ZnO(R-Si-e)); 183 nm, 2.5 µm (ZnO(S-1)), and 227 nm, 1.2 µm (ZnO(S-2)). On a sub-micron scale, the ZnO(R-Si) with TEOS modification shows the finest dispersion with the lowest average sizes, while ZnO(S-1) shows the largest aggregates. The ZnO(S-2) without modification also has low aggregate sizes but has the largest average diameter (see Supplementary Materials, Figure S3). The higher average size of ZnO(S-2) can also be related to the rather broad size distribution observed in TEM (Figure 1e, Table 2). While some aggregates are apparent for all ZnO types, they are generally homogeneously distributed throughout the matrix with a relatively low size. The silane surface treatment of ZnO with TEOS (ZnO(R-Si)) and MPS (ZnO(R-Si-e)) should improve the compatibility with the more hydrophobic PHBHHx polymer matrix, resulting in a finer NP dispersion as similarly demonstrated in PLA nanocomposites [51]. In conclusion, the TEOS surface treatment of ZnO(R-Si) results in the finest dispersion in the PHBHHx matrix due to increased compatibility with a hydrophobic PHBHHx matrix.

### 3.2. Chemical Properties

The chemical properties of ZnO NPs, PHBHHx, and their nanocomposites are investigated with ATR-FTIR because ZnO NPs can influence the crystallization of polymers by chemical interaction. The interaction between ZnO NPs and the polymer matrix highly depends on the chemical structure of both, generally including hydrogen bonding between available hydroxyl (-OH) groups on the surface of the filler and the ester carbonyl groups (C=O) in PHAs [56]. However, the presence of hydroxyl groups at the ZnO surface can be altered by oxygen vacancies absorbing -OH groups [25], or specific ethanol-assisted production methods that increase the number of -OH groups [57].

**Figure 3:**
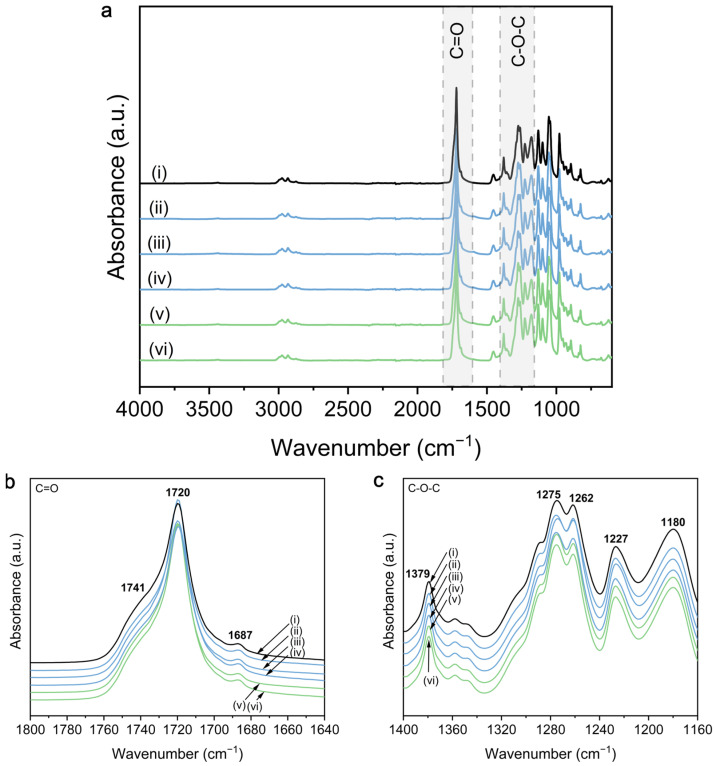
FTIR spectra of PHBHHx/ZnO nanocomposites with different types of ZnO NP at a concentration of 5 wt.%, including (i) pure PHBHHx, (ii) ZnO(R), (iii) ZnO(R-Si), (iv) ZnO(R-Si-e), (v) ZnO(S-1), (vi) ZnO(S-2), with (**a**) overview of FTIR spectra, (**b**) detail of the C=O region (1800 to 1640 cm^−1^), and (**c**) detail of the C-O-C region (1400 to 1160 cm^−1^).

The FTIR spectra of PHBHHx and 5 wt.% ZnO nanocomposites are shown in Figure 3. The detailed reference spectrum of PHBHHx can be found in Supplementary Materials, Figure S4. The spectra of PHBHHx and ZnO nanocomposites are mainly characterized by an ester function at around 1720 cm^−1^ (Figure 3a,b) and C-O-C stretching in the region between 1150 and 1500 cm^−1^ (Figure 3c). Changes in the ester region (1720 cm^−1^) can be indicative of hydrogen bonding interactions between PHBHHx and ZnO NPs. Figure 3b indicates that there is no hydrogen bond formation between PHBHHx and any of the ZnO types because no shifts in intensity and wavenumber of the C=O stretching are apparent. To compare, hydrogen bonding in PHA/ZnO nanocomposites has been shown for PHB [35], PHBV [37], and PHBHHx [46,58], but also for other NPs like graphene oxide (GO) [59]. Here, the ester function for PHBHHx/ZnO at 1720 cm^−1^ appears wider, at a lower wavenumber and/or with increased intensity compared to the neat polymer and is ascribed to the availability of -OH groups on the surface of ZnO to engage in H-bonding. A possible explanation for the absence of hydrogen bonding between PHBHHx and different types of ZnO in this study is the rather low amount of -OH groups on the surface of ZnO, as confirmed by FTIR and TGA (Supplementary Materials, Figures S5 and S6 and Table S6).

The characteristic surface properties of commercial ZnO NPs used in this work are assumed to be related to the industrial production process, including heat treatments (annealing) or calcination to ensure a high NP purity, crystallinity, and stability. The latter is known to reduce or even remove the majority of functional groups (including -OH) from the ZnO NP surface [60,61]. It has also been shown that heat-treated ZnO NPs contain only very small amounts of -OH groups, even after exposure to humidity [62]. Bressy et al. [48] showed that the hydroxyl density (OH/nm^2^) for a commercial ZnO was ~9- to 32-times lower compared to their as-prepared ZnO NPs. Alternatively, the absence of H-bond formation could also be explained by the structure of PHBHHx, as it has been shown that hydrogen interactions between -OH groups of, e.g., fillers and PHBHHx, is weaker compared to PHB due to the steric hindrance of longer 3-HHx side chains [63]. In addition, the relatively high crystallinity of PHBHHx in this study could further reduce the tendency for H-bonding, as it has been suggested that H-bonding occurs in the amorphous region of the C=O groups rather than in crystalline [63]. Silva et al. showed the appearance of a new shoulder in the C-O-C region at 1083 cm^−1^ for PHB/ZnO nanocomposites, indicative of a chemical interaction between ZnO and PHB [31]. In accordance with our results, this peak does not appear in the PHBHHx/ZnO nanocomposites for different ZnO types and concentrations (Supplementary Materials, Figure S7).

Both C=O and C-O-C spectral regions can be used for a qualitative analysis of the crystallinity of PHBHHx/ZnO films. All presented FTIR spectra are baseline-corrected and normalized to the 1453 cm^−1^ band, which is considered to be insensitive to changes in crystallinity [64]. Shifts in intensity and positions of the C=O region (1740–1720 cm^−1^) can be related to changes in polymer crystallinity because the carbonyl band of PHBHHx contains distinct but overlapping spectral features from both crystalline and amorphous phases [65]. A detailed calculation of the qualitative crystallinity fraction (*X_C,FTIR_*) is shown in Supplementary Materials S8, indicating an increase from *Xc,_FTIR_* = 77.1% for PHBHHx up to a maximum of *Xc_,FTIR_* = 85.5% for ZnO(S-1). The difference in crystallinity following the indices I_1227_/I_1453_ and I_1379_/I_1180_ is much smaller but also generally higher for the PHBHHx/ZnO nanocomposites.

In conclusion, no direct chemical interactions between PHBHHx and any of the commercial ZnO types were detected, independent of ZnO surface treatment or morphology. This is in line with the low amount of hydroxyl groups at the ZnO NP surfaces in combination with a relatively high degree of crystallinity of PHBHHx. The FTIR measurements indicate a slightly higher crystallinity for all PHBHHx/ZnO nanocomposites, not depending on the ZnO shape, size, or surface treatment.

### 3.3. Thermal Properties

**Figure 4:**
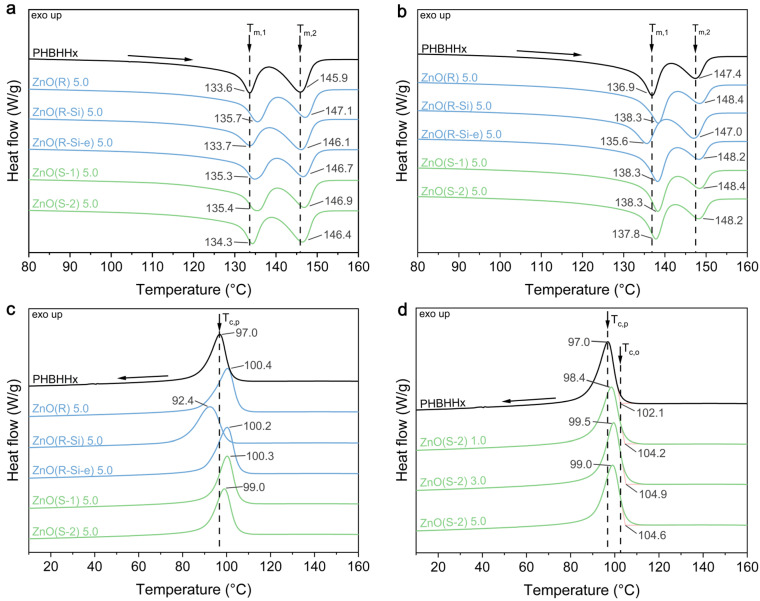
Non-isothermal DSC curves for neat PHBHHx and ZnO nanocomposites showing (**a**) first heating cycle for 5 wt.% ZnO nanocomposites, (**b**) second heating cycle for 5 wt.% ZnO nanocomposites, (**c**) first cooling cycle for 5 wt.% ZnO nanocomposites, (**d**) cooling cycle for different concentrations (1, 3, and 5 wt.%) of ZnO(S-2) nanocomposites. The curves are averaged (*n* = 2), and the denoted values are in degrees Celsius (°C). The arrows indicate the heating (up) or cooling (down) during DSC measurements.

The influence of ZnO NPs on the melting and crystallization of PHBHHx was investigated with DSC. The heating and cooling scans of the PHBHHx/ZnO nanocomposite films for different ZnO types and concentrations are shown in Figure 4 and the characteristic values are shown in Table 3 and Table 4. The first and second heating scans (Figure 4a,b) and Table 3 show that the melting behavior of the PHBHHx/ZnO films is clearly defined by the multiple melting peaks of PHBHHx [66]. The first endothermic peak at T_m,1_ originates from the melting of primary crystals formed during the initial crystallization during processing (first heating in Figure 4a) or at the single crystallization temperature (T_c_) (second heating in Figure 4b) [67,68]. The second endothermic peak at T_m,2_ is due to the melting of crystals formed by reorganization or thickening while heating in the DSC run [66,69]. In this way, only one well-defined crystallization peak (Figure 4c) corresponds with two endothermic melting peaks. In addition, the DSC curves of pure ZnO NP did not reveal any significant endothermic or exothermic events in the temperature range of −30 to 170 °C, as expected in agreement with previous literature [70]. Some changes in the heating cycles are apparent for the PHBHHx/ZnO nanocomposites. The T_m,1_ and T_m,2_ in the first and second heating cycles increase slightly for all ZnO types except for ZnO(R-Si), compared to neat PHBHHx. However, no significant trend of ZnO type on the crystallinity (*X_C,DSC_*) and melting enthalpy (ΔH_m_) is observed from DSC.

The first cooling scan (Figure 4c,d, Table 4) shows a single and well-defined crystallization peak [71] at a temperature of T_c,p_ = 97 °C for PHBHHx, which slightly shifts to higher temperatures for all PHBHHx/ZnO nanocomposites with increasing ZnO concentration, except for ZnO(R-Si). The nanocomposites with ZnO(R-Si) exhibit lower T_c,p_, and crystallization rates (R), indicating slower crystallization from the melt. A more difficult crystallization was also observed for PLA nanocomposites with silane surface-treated ZnO NPs, and attributed to strong interactions between the silane group and carbonyl functions of PLA [72].

In conclusion, the DSC results indicate that there is no direct correlation between ZnO shape and size on the crystallization and melting behavior of PHBHHx. On the contrary, silane modification (TEOS) of ZnO reduces the crystallization rate from the melt and slightly reduces the crystal perfection of PHBHHx, despite the finest dispersion (SEM, Figure 2b). The incorporation of ZnO does not lead to an increase in PHBHHx crystallinity, as supported by WAXD measurements (see Supplementary Materials S9), also showing that sizes of the PHBHHx unit cells are unaffected by the incorporation of all ZnO types.

**Figure 5:**
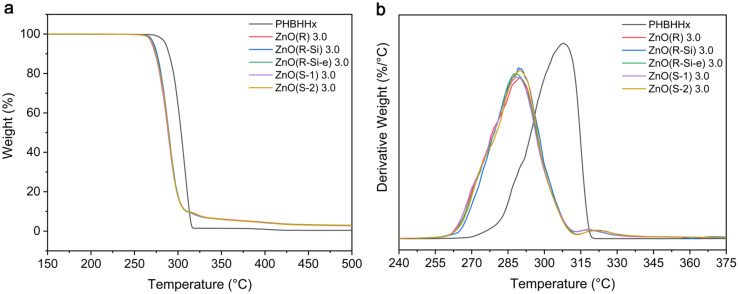
TGA curves of 3 wt.% PHBHHx/ZnO nanocomposites, showing (**a**) the weight loss curves and (**b**) the derivative weight loss curves in function of the temperature.

The influence of ZnO NPs on the thermal stability of PHBHHx was investigated with TGA measurements. An overview of the TGA curves for PHBHHx/ZnO with 3 wt.% ZnO loading is shown in Figure 5 and characteristic parameters for all ZnO types and concentrations are summarized in Supplementary Materials, Table S10. TGA curves for all ZnO NP types are shown in Supplementary Materials, Figure S6.

A single degradation step takes place for PHBHHx starting around 292 °C with a maximum degradation rate of around 308 °C. The decomposition process of PHBHHx is similar to that of PHB and PHBV, following a random chain scission mechanism of the ester groups [73,74,75]. The nanocomposites follow the same single degradation step as PHBHHx, as shown in the derivative curves (Figure 5b). The thermal decomposition of all PHBHHx/ZnO nanocomposites occurs at lower temperatures compared to the neat PHBHHx, with no clear differences for the various ZnO types. The decrease in the temperature at the maximum degradation rate could be explained by the catalytic effect and high heat conductivity of ZnO, which was also reported for PHBHHx/ZnO [39] and PHBV/ZnO [27]. A proposed mechanism for the thermal degradation of PHBV/ZnO was the formation of zinc salts via a reaction of polymeric carboxylic groups with Zn-OH, accelerating the degradation process [28]. However, the reduction in thermal stability towards 285 °C should not influence the melt-processing of PHBHHx/ZnO compounds because the processing temperature is significantly lower (~165 °C).

In contrast to our results, ZnO NPs can also improve the thermal stability of PHA/ZnO nanocomposites due to the high thermal conductivity of the ZnO NPs that facilitate heat dissipation in the polymer matrix [35,37,76], the barrier effect of ZnO NPs that restrict the transport of polymer decomposition products to the gas phase [76], and strong hydrogen bond interactions between the filler and matrix that boost this barrier effect [35,37,49].

### 3.4. Mechanical Properties

**Figure 6:**
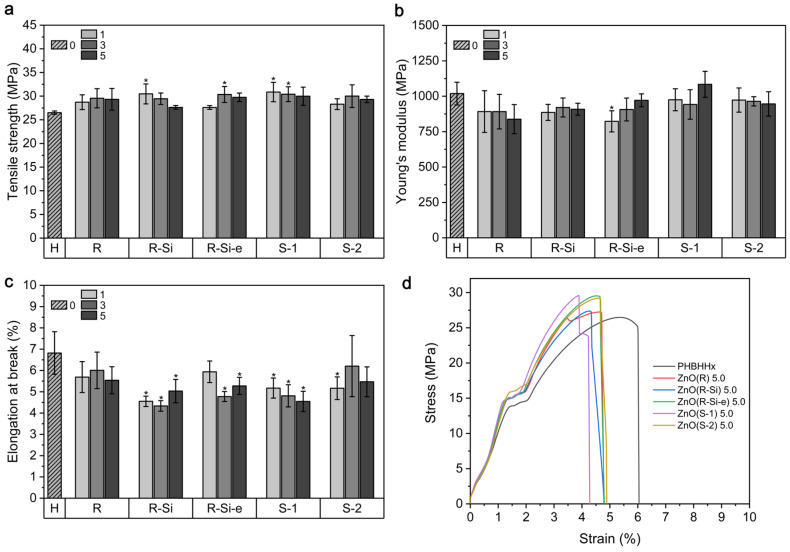
Mechanical properties of PHBHHx/ZnO nanocomposite films with different ZnO types and concentrations (1, 3, and 5 wt.%) and unfilled PHBHHx (H) films (*n* = 5, ±1 SD): (**a**) tensile strength, (**b**) Young’s modulus, (**c**) elongation at break, (**d**) average stress-strain curves for 5 wt.% ZnO. Bars with * denote statistical differences (*p* < 0.05) compared to PHBHHx.

The mechanical properties tensile strength (σ), Young’s modulus (E), and elongation at break ($\varepsilon_{b}$) of the PHBHHx/ZnO films together with the averaged stress-strain curves for 5 wt.% ZnO are shown in Figure 6. The PHBHHx films show a σ of 26.5 ± 0.4 MPa and an increasing trend is apparent with the incorporation of ZnO NPs, with a maximum average σ of 30.9 ± 2.1 MPa and 30.5 ± 2.1 MPa for 1 wt.% ZnO(S-1) and 1 wt.% ZnO(R-Si), respectively. These are improvements in σ of 16.6% and 15.0%, respectively. However, the σ is independent of the ZnO concentration and type and only some samples show a statistically significant difference with PHBHHx (Figure 6a). The E of PHBHHx is 1019 ± 80 MPa and the ZnO nanocomposites show a rather decreasing trend of E, but with only one sample type that is statistically lower (1 wt.% ZnO(R-Si-e)), with an E of 823 ± 75 MPa (Figure 6b). This is a reduction of 19.2%. Further, the $\varepsilon_{b}$ of PHBHHx is around 6.8 ± 1.0%, and a decreasing trend of $\varepsilon_{b}$ is apparent with the incorporation of ZnO. The $\varepsilon_{b}$ drops below a value of ~5.5% and is statistically lower for ZnO(R-Si), ZnO(R-Si-e), and ZnO(S-1), except for 1 wt.% ZnO(R-Si-e). A minimum $\varepsilon_{b}$ is measured for 3 wt.% ZnO(R-Si), with a reduction of 36.8% compared to unfilled PHBHHx.

In unfilled polymer systems, the mechanical properties σ and E are generally improved at higher crystallinity values. The crystallinity of polymers can be increased after the incorporation of NPs when acting as nucleating agents. However, our results from DSC and XRD (Supplementary Materials S9) show that the crystallinity of all nanocomposite films remains in the same range as the neat PHBHHx, which can be correlated with a low effect of ZnO incorporation on the mechanical properties of PHBHHx. In addition, the crystallinity values from FTIR indicated only minor variations in X_C_ upon the addition of ZnO (Supplementary Materials S8). Nevertheless, it has been previously shown that the mechanical properties of filled PHB systems cannot be clearly correlated to the degree of crystallinity and depend highly on the polymer-filler system under study [77].

Moreover, no clear trends in mechanical properties for different ZnO types were revealed. Due to the absence of strong matrix-filler interactions, this study mainly demonstrates the direct influence of ZnO morphology and their specific stress concentrations (induced by, e.g., shape) on the mechanical behavior. The additional surface functionalization did not improve the mechanical properties, despite the finer dispersion quality of, e.g., ZnO(R-Si). On the other hand, the results highlight that the mechanical properties and especially the flexibility of PHBHHx can be retained and do not worsen when incorporating different ZnO NPs up to concentrations of at least 5 wt.%.

### 3.5. UV Barrier Properties

**Figure 7:**
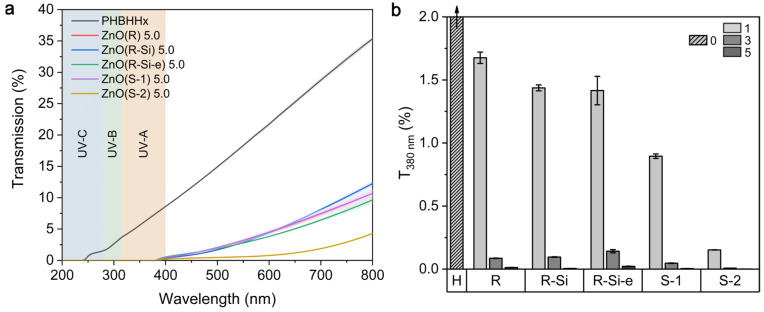
Evaluation of UV barrier properties of PHBHHx/ZnO nanocomposite films with different ZnO types and concentrations (1, 3, and 5 wt.%) and unfilled PHBHHx (H) films, (**a**) UV–VIS spectra for films with 5 wt.% ZnO of different types, (**b**) transmission at a wavelength of 380 nm (UV-A). The curves are averaged (*n* = 3) and the shaded colors in (**a**) and error bars in (**b**) represent 1 SD.

UV/VIS measurements were performed to study the UV barrier effect of the PHBHHx/ZnO nanocomposite films. The UV/VIS transmission spectra of the PHBHHx and PHBHHx/ZnO films with 5 wt.% ZnO are shown in Figure 7a. The UV/VIS spectra for 1 and 3 wt.% ZnO are shown in Supplementary Materials S11. These results show that the PHBHHx/ZnO films have significantly reduced UV transmission in the wavelength range 250 nm to 380 nm, and thus they can effectively act as a UV barrier. The ZnO NPs exhibit an absorption peak between 370 nm and 390 nm, and the UV barrier in the UVA wavelength region is highly concentration-dependent and increases with ZnO concentration (Figure 7b).

Similar UV barrier effects were also observed for ZnO NP ultrasonic spray-coated PHBHHx [78]. The UV barrier effect of ZnO NPs can be explained by the combination of bandgap absorption and light scattering [79]. Nano-sized particles have a large surface area to volume ratio that results in a high UV-blocking effect observed for the prepared nanocomposites, mainly expressed at high concentrations of 5 wt.%. The steady reduction in transmission within present concentration ranges is in line with good dispersion of the ZnO in the PHBHHx matrix. The UV-barrier effect is higher for the spherical NPs (ZnO(S-1) and ZnO(S-2)) and similar for the rod-like NPs (ZnO(R), ZnO(R-Si), and ZnO(R-Si-e)). The increased UV barrier effect for ZnO(S-2) can be correlated to the lower transmission in the visible wavelengths (Figure 7a). This higher opacity for ZnO(S-2) can be related to the higher average ZnO size in PHBHHx, as shown in SEM images (Figure 2e and Supplementary Materials S3).

### 3.6. Colorimetric Properties and Opacity

The color and opacity of the nanocomposite films are important for practical packaging applications because they influence the connection between the consumer and the food product. The combination of extrusion and compression molding results in homogeneous and translucent PHBHHx/ZnO films as shown in Supplementary Materials, Figure S12. The thickness of the PHBHHx and PHBHHx/ZnO films was on average 180 ± 10 µm. The L*, a*, b* colorimetric parameters, together with the total color difference ∆E, are shown in Table 5. The nanocomposite films appear slightly whiter (higher L*) and greener (lower a*) compared to PHBHHx. The nanocomposite films undergo yellowing (higher (b*) and the total color difference increases with ZnO concentration, except for ZnO(R-Si).

**Figure 8:**
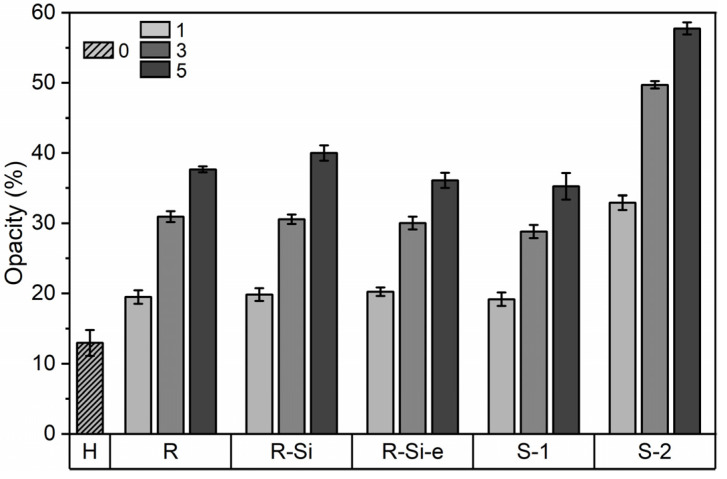
Opacity measurements of PHBHHx/ZnO nanocomposite films (*n* = 8, ±1 SD) with different ZnO types and concentrations (1, 3, and 5 wt.%) and unfilled PHBHHx (H) films.

The opacity (Y) of the nanocomposite films is shown in Figure 8. The opacity gradually increases with ZnO concentration, and the opacity values are similar for all ZnO types, except for ZnO(S-2). The opacity of the ZnO(S-2) composites is higher for all concentrations, similar to a reduced transmission in the visible wavelengths (Figure 7a and Supplementary Materials, Figure S11). This could be attributed to the higher average diameter of the ZnO(S-2) NPs as determined via SEM (Figure 2e and Supplementary Materials, Figure S3) in PHBHHx and via TEM (Figure 1e and Table 2) before incorporation in PHBHHx. Following the Rayleigh law, the intensity of scattered light is higher with increasing particle diameter, resulting in a more turbid (opaque) appearance of the nanocomposite [80]. The higher opacity in the ZnO(S-2) samples compared to the other ZnO types is not attributed to a higher ZnO loading, as confirmed by similar residue (R) values in TGA measurements (Supplementary Materials, Table S10). Despite the opacity increases with ZnO loading, the films remain translucent, as shown by the example images in Supplementary Materials, Figure S12.

### 3.7. Wetting Properties

**Figure 9:**
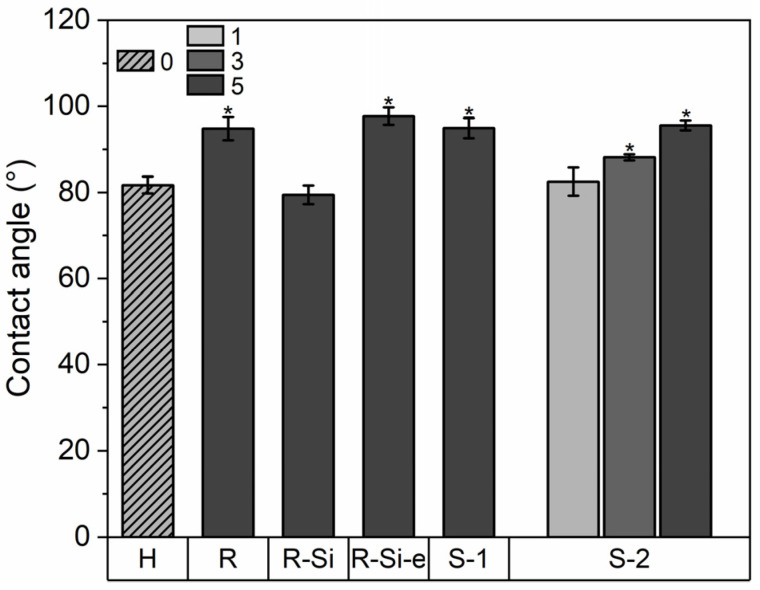
Water contact angle of PHBHHx/ZnO nanocomposite films (*n* = 5, ±1 SD) with different ZnO types and concentrations (1, 3, and 5 wt.%) and unfilled PHBHHx (H) films. Bars with a * denote statistical differences (*p* < 0.05) compared to PHBHHx.

The wetting behavior of food packaging materials is important because hydrophobic surfaces can improve the resistance to chemical interactions with food and moisture and can even have antibacterial effects, leading to increased food freshness [81]. The water contact angles of the PHBHHx/ZnO nanocomposite films are shown in Figure 9. The PHBHHx film has a contact angle of around 82 ± 2° and it increases towards a maximum of 98 ± 2° for 5 wt.% ZnO(R-Si-e). Statistical analysis shows that all ZnO NPs significantly increase the contact angle of PHBHHx with a ZnO loading above 3 wt.%. Surprisingly, this is not the case for 5 wt.% of the hydrophobic type ZnO(R-Si). This could indicate that the surface treatment of the ZnO changes the dispersion state of the NPs at the surface of the nanocomposite. A possible explanation is that the finer dispersion of ZnO(R-Si) creates a smoother surface that can result in a lower contact angle, compared to the other ZnO types with larger sizes (in combination with agglomerations) [82]. In addition, the contact angle of PHBHHx/ZnO is concentration-dependent, increasing with ZnO concentration, as shown for ZnO(S-2).

### 3.8. Gas Permeability Properties

A major criterion for food packaging materials is the barrier properties for gases such as oxygen. The oxygen permeability coefficients for neat versus PHBHHx/ZnO nanocomposite films are shown in Table 6. PHBHHx has a PO_2_ of 3.7 cm³ mm/m² day, which decreases upon loading of ZnO(R) and ZnO(S-2) but increases upon loading of ZnO(R-Si). The maximum decrease of PO_2_ amounts to 36% for a low loading of 1 wt.% ZnO(S-2). The decreased oxygen permeability of the nanocomposites is likely related to the fine dispersion of ZnO NPs that increases the tortuosity path of the oxygen molecules throughout the polymer matrix. Surprisingly, the PO_2_ of PHBHHx/ZnO(R-Si) is increased, while it has the finest dispersion. This is possibly due to specific interactions between ZnO(R-Si) and PHBHHx, as evidenced by a decrease in the crystallization temperature as shown before (Figure 4c). In general, an increase in PHA crystallinity results in reduced gas permeability values [83]. However, the ZnO(R-Si) nanocomposites only show a little lower crystallinity compared to ZnO(S-2) and ZnO(R), which would not explain the increased oxygen permeability.

In this study, however, the reduced PO_2_ of PHBHHx/ZnO nanocomposites cannot be explained by the formation of a physical crosslinked network due to hydrogen bond formation between PHBHHx and ZnO as previously suggested in [49], owing to the absence of H-bond interactions as shown by FTIR measurements in previous paragraphs (Figure 3).

### 3.9. Antibacterial Properties

To assess the antibacterial potential of the ZnO types used in nanocomposite films, MIC tests were performed for *E. coli* and *S. aureus* (Supplementary Materials S13). It is clear from the MIC results that all ZnO NPs have a higher antibacterial effect against *S. aureus* compared to *E. coli.* The ZnO(S-2) type has the lowest MIC value of 416.7 µg/mL for *S. aureus* and it is expected to exhibit the highest antibacterial effect when incorporated in PHBHHx. Smaller ZnO NP sizes generally have higher antibacterial activity [84], but it has been shown that high porosity in ZnO NPs can also increase the antibacterial effect [85]. The higher pore volume of ZnO(S-2) compared to other ZnO NPs (Table 2) might explain the higher antibacterial activity.

**Figure 10:**
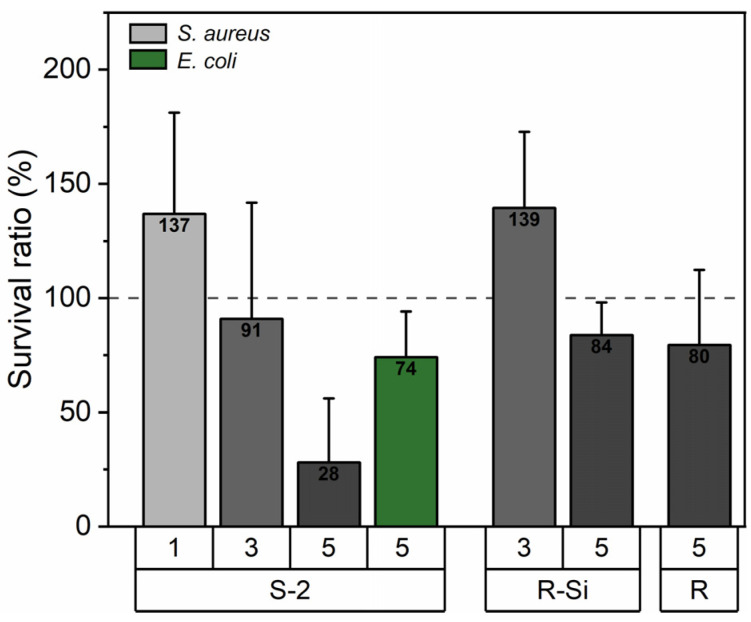
Survival ratio (SR) of *S. aureus* and *E. coli* when incubated on PHBHHx/ZnO nanocomposite films with a ZnO concentration of 1, 3, or 5 wt.%. The survival ratio of the bacteria on PHBHHx was 100% (dashed line).

The antibacterial properties of the PHBHHx/ZnO nanocomposite films against the bacteria *E. coli* and *S. aureus* are shown in Figure 10. These results show the survival ratio of *E. coli* and *S. aureus* on the surface of the PHBHHx/ZnO nanocomposites. The survival ratio for *S. aureus* shows a decreasing trend with ZnO loading and is effectively reduced to 28% for 5 wt.% ZnO(S-2). The other ZnO types show a smaller decrease in the survival ratio for *S. aureus.* The incorporation of 5 wt.% ZnO(S-2) also reduces the survival ratio of *E. coli* to around 74%. The higher antibacterial effect of ZnO(S-2) is in accordance with the observed MIC assessment, indicating higher activity for the particles with a higher pore volume.

Alternatively, the silane treatment of the ZnO(R-Si) does not result in improved antibacterial activity, while other studies reported that silane (TEOS)-modified ZnO (3% loading) showed better antibacterial effect against gram-positive (*S. aureus*) and gram-negative (*Klebsiella pneumonia*) bacteria compared to untreated ZnO in melt-spun PLA nanocomposite yarns [86]. The better dispersion state of silane-treated ZnO could generally promote the photocatalytic effect and production of OH· radicals, which was found to be the main antibacterial factor of the NPs (in contrast to the release of Zn^2+^ ions). The opposite results were also reported, where untreated ZnO NPs showed better antibacterial activity to gram-positive and gram-negative bacteria compared to silane (TEOS)-treated ZnO NPs in melt-processed polyolefin nanocomposites (2% ZnO loading) [50]. However, the antibacterial effect of silane-treated ZnO was still effective against the gram-positive *S. aureus*. Based on our study, no direct correlation between the surface hydrophobicity of PHBHHx/ZnO films and the antibacterial activity could be established, indicating that the intrinsic mechanisms for the antibacterial effects of ZnO indeed determine the antibacterial effects of the nanocomposites in relation to the intrinsic nanoparticle morphology.

## 4. Conclusions

In this study, the influence of commercial ZnO NPs with different shapes, sizes, and surface treatments on the structure–property relationships of melt-processed PHBHHx/ZnO nanocomposite films was investigated. The findings indicate that the chemical interactions (e.g., hydrogen bonding) between ZnO NPs and PHBHHx do not depend on size, shape, or silane surface treatment of ZnO, but rather on the availability of hydroxyl groups on the NP surface and on the polymer characteristics (such as crystallinity). All ZnO types showed a rather homogeneous distribution in PHBHHx up to concentrations of 5 wt.%, but ZnO with triethoxy caprylyl silane surface modification showed a finer dispersion (smallest average ZnO size). However, we showed that the functional characteristics, such as the interplay between crystallization, mechanical, UV, and gas barrier properties are not directly dictated by the ZnO dispersion quality, nor by the ZnO shape or surface treatment. The antibacterial properties of the nanocomposites seem to be dictated by the intrinsic antibacterial mechanisms of the ZnO NPs and are not significantly influenced by dispersion state, ZnO surface treatment, or surface wetting behavior.

It was confirmed that PHBHHx remains flexible (i.e., without modification of the processability and crystallization properties) upon ZnO loading (5 wt.%) and that the appropriate selection of the ZnO type can enhance the functional properties of PHBHHx such as UV and gas barrier combined with antibacterial effects.

## Figures and Tables

**Table 1:** Overview of the used commercial ZnO NPs.

Abbreviation	Commercial Name	Morphology	Surface Modification
ZnO(R)	Tenray Z2	Rod	None
ZnO(R-Si)	Tenray Z2P	Rod	triethoxy caprylyl silane (TEOS)
ZnO(R-Si-e)	Tenray Z2E	Rod	3-(methacryloyloxy)propyltrimethoxy-silane (MPS)
ZnO(S-1)	Nanosun P99/30, NM-112	Spherical	None
ZnO(S-2)	ZnO no. 544906	Spherical	None

**Table 2:** Physicochemical properties of the ZnO NP types.

Parameter	ZnO(R)	ZnO(R-Si)	ZnO(R-Si-e)	ZnO(S-1)	ZnO(S-2)
Shape	Rod	Rod	Rod	Spherical	Spherical
TEM size L/D (SD) or D (SD) (nm)	61(20)/24(11)	60(20)/15(6)	60(21)/18(8)	36(9)	79(62)
Surface area (BET *, m^2^/g)	34	42	41	30	17
Volume of pores (BJH **, cm^3^/g)	0.065	0.060	0.060	0.054	0.229
Pore size (BJH, 4 V/A, nm)	5.5	5.3	5.4	5.9	1.9
Hydrophobicity (CA, °)	11	>160	15	<10	15

* Brunauer, Emmett and Teller (BET) Method, ** Barrett, Joyner and Halenda (BJH) method.

**Table 3:** Non-isothermal DSC parameters during the first and second heating cycles of PHBHHx and the ZnO nanocomposites with different ZnO concentrations of 1, 3, and 5 wt.% (*n* = 2, ±1 SD).

Sample	C_ZnO_ (wt.%)	First Heating			Second Heating		
		T_m,1_ (°C)	T_m,2_ (°C)	*X_C,DSC_* (%)	T_m,1_ (°C)	T_m,2_ (°C)	ΔH_m_ (J/g)
PHBHHx	0	133.6 ± 0.2	145.9 ± 0.1	55.1 ± 0.0	136.9 ± 0.2	147.4 ± 0.0	51.4 ± 0.1
ZnO(R)	1	134.7 ± 1.2	146.6 ± 0.5	56.0 ± 0.0	137.7 ± 0.0	148.1 ± 0.0	50.4 ± 0.6
	3	135.1 ± 1.4	146.8 ± 0.7	56.2 ± 1.2	138.0 ± 0.4	148.2 ± 0.1	51.4 ± 0.8
	5	135.7 ± 1.0	147.1 ± 0.1	56.1 ± 0.1	138.3 ± 0.1	148.4 ± 0.2	50.4 ± 0.5
ZnO(R-Si)	1	132.8 ± 1.0	145.7 ± 0.4	53.9 ± 0.0	135.7 ± 0.0	147.1 ± 0.0	50.8 ± 0.2
	3	133.1 ± 1.1	146.0 ± 0.5	54.1 ± 0.5	136.2 ± 0.1	147.5 ± 0.1	50.2 ± 0.8
	5	133.7 ± 0.3	146.1 ± 0.2	54.3 ± 0.3	135.6 ± 0.5	147.0 ± 0.1	51.1 ± 0.1
ZnO(R-Si-e)	1	134.7 ± 1.3	146.7 ± 0.4	54.5 ± 1.5	137.9 ± 0.2	148.3 ± 0.2	50.7 ± 0.5
	3	135.0 ± 1.4	146.8 ± 0.5	53.4 ± 0.7	138.2 ± 0.2	148.3 ± 0.2	49.6 ± 1.6
	5	135.3 ± 1.4	146.7 ± 0.7	55.5 ± 0.4	138.3 ± 0.0	148.2 ± 0.0	51.5 ± 0.4
ZnO(S-1)	1	134.1 ± 0.2	146.3 ± 0.0	55.8 ± 0.5	137.8 ± 0.0	148.1 ± 0.0	51.1 ± 0.2
	3	135.2 ± 1.2	146.8 ± 0.6	55.9 ± 0.0	137.9 ± 0.1	148.3 ± 0.1	50.6 ± 0.2
	5	135.4 ± 1.3	146.9 ± 0.5	54.4 ± 2.1	138.3 ± 0.0	148.4 ± 0.0	51.1 ± 0.3
ZnO(S-2)	1	135.5 ± 1.4	146.8 ± 0.4	56.2 ± 0.5	137.7 ± 0.0	148.0 ± 0.1	49.7 ± 0.7
	3	135.3 ± 1.5	146.8 ± 0.6	55.4 ± 0.7	138.0 ± 0.1	148.2 ± 0.0	50.3 ± 0.1
	5	134.3 ± 0.5	146.4 ± 0.2	55.7 ± 0.5	137.8 ± 0.1	148.2 ± 0.0	51.5 ± 0.2

**Table 4:** Non-isothermal DSC parameters during the first cooling cycle of PHBHHx and the ZnO nanocomposites with different ZnO concentrations of 1, 3, and 5 wt.% (*n* = 2, ±1 SD).

Sample	C_ZnO_ (wt.%)			First Cooling		
		T_c,o_ (°C)	T_c,p_ (°C)	ΔH_c_ (J/g)	t_1/2_ (min)	R (min^−1^)
PHBHHx	0	102.1 ± 0.8	97.0 ± 0.5	47.0 ± 0.4	0.990 ± 0.024	1.01 ± 0.02
ZnO(R)	1	104.3 ± 0.3	98.7 ± 0.0	47.5 ± 0.0	0.903 ± 0.014	1.11 ± 0.01
	3	105.2 ± 0.7	99.7 ± 1.1	48.5 ± 0.7	0.858 ± 0.087	1.17 ± 0.06
	5	105.9 ± 0.2	100.4 ± 0.2	48.9 ± 0.3	0.832 ± 0.031	1.20 ± 0.02
ZnO(R-Si)	1	99.1 ± 0.0	93.6 ± 0.2	47.7 ± 0.5	1.177 ± 0.014	0.85 ± 0.01
	3	100.3 ± 0.3	94.6 ± 0.2	48.2 ± 0.4	1.102 ± 0.012	0.91 ± 0.00
	5	99.8 ± 0.8	92.4 ± 1.8	48.3 ± 0.4	1.208 ± 0.064	0.83 ± 0.02
ZnO(R-Si-e)	1	104.0 ± 0.3	98.7 ± 0.3	47.2 ± 0.5	0.902 ± 0.017	1.11 ± 0.01
	3	104.9 ± 0.2	99.3 ± 0.4	46.8 ± 0.2	0.907 ± 0.071	1.11 ± 0.04
	5	105.7 ± 0.1	100.2 ± 0.3	49.2 ± 0.2	0.830 ± 0.024	1.21 ± 0.02
ZnO(S-1)	1	104.5 ± 0.4	99.0 ± 0.4	47.4 ± 0.7	0.868 ± 0.031	1.15 ± 0.02
	3	104.9 ± 0.4	99.4 ± 0.3	48.1 ± 0.5	0.873 ± 0.014	1.15 ± 0.01
	5	105.7 ± 0.2	100.3 ± 0.2	48.0 ± 0.1	0.812 ± 0.021	1.23 ± 0.02
ZnO(S-2)	1	104.2 ± 0.0	98.4 ± 0.1	46.3 ± 0.5	0.915 ± 0.017	1.09 ± 0.01
	3	104.9 ± 0.3	99.5 ± 0.2	46.7 ± 0.2	0.853 ± 0.014	1.17 ± 0.01
	5	104.6 ± 0.5	99.0 ± 0.4	47.6 ± 0.5	0.875 ± 0.031	1.14 ± 0.02

**Table 5:** Colorimetric properties (L*a*b*) of PHBHHx/ZnO nanocomposite films (*n* = 10, ±1 SD).

Sample	C_ZnO_ (wt.%)	L*	a*	b*	∆E
PHBHHx	0	94.4 ± 0.1	−0.9 ± 0.1	4.8 ± 0.4	/
ZnO(R)	1	94.8 ± 0.1	−1.0 ± 0.1	4.9 ± 0.3	0.37
	3	94.8 ± 0.1	−1.0 ± 0.0	5.9 ± 0.2	1.10
	5	94.5 ± 0.1	−1.1 ± 0.0	7.8 ± 0.3	3.01
ZnO(R-Si)	1	95.0 ± 0.1	−0.8 ± 0.1	3.7 ± 0.3	1.25
	3	95.4 ± 0.1	−0.9 ± 0.0	3.8 ± 0.2	1.43
	5	95.3 ± 0.1	−1.2 ± 0.0	5.6 ± 0.3	1.13
ZnO(R-Si-e)	1	94.8 ± 0.1	−1.0 ± 0.0	5.2 ± 0.2	0.51
	3	95.0 ± 0.1	−1.1 ± 0.1	5.5 ± 0.3	0.87
	5	95.0 ± 0.1	−1.1 ± 0.0	5.8 ± 0.2	1.15
ZnO(S-1)	1	94.8 ± 0.1	−1.1 ± 0.1	5.2 ± 0.2	0.54
	3	94.7 ± 0.1	−1.1 ± 0.0	6.5 ± 0.2	1.74
	5	94.6 ± 0.0	−1.1 ± 0.0	7.2 ± 0.2	2.36
ZnO(S-2)	1	95.1 ± 0.1	−1.1 ± 0.0	5.3 ± 0.2	0.85
	3	95.3 ± 0.1	−1.1 ± 0.0	5.8 ± 0.2	1.31
	5	94.7 ± 0.9	−1.2 ± 0.1	7.9 ± 0.4	3.06

**Table 6:** Gas permeability properties (PO_2_) of PHBHHx/ZnO nanocomposite films with a ZnO concentration of 1, 3, or 5 wt.%.

Sample	ZnO Concentration (wt.%)	PO_2_	
		(cm³ mm/m² day)	% Change
PHBHHx	0	3.67 ± 0.11	Reference
ZnO(R)	5	3.20 ± 0.06	−13
ZnO(R-Si)	5	4.30 ± 0.23	+17
ZnO(S-2)	1	2.35 ± 0.15	−36
	3	2.84 ± 0.25	−23
	5	2.71 ± 0.01	−26

## Data Availability

The original contributions presented in the study are included in the article/Supplementary Material, further inquiries can be directed to the corresponding authors.

## Supplementary Materials

The following supporting information can be downloaded at: https://www.mdpi.com/article/10.3390/polym16131861/s1, Figure S2: Exemplary ImageJ analysis of TEM images from ZnO NPs: (a) ZnO(R), (b) ZnO(R-Si), (c) ZnO(R-Si-e), (d) ZnO(S-1), and (e) ZnO(S-2). The yellow lines indicate measurements of ZnO NP rod lengths or diameters, depending on ZnO morphology (rod vs. spherical morphology).; Figure S3: SEM size distribution analysis for PHBHHx/ZnO nanocomposites with different ZnO types at a concentration of 5 wt.%.; Figure S4: Detailed FTIR spectrum of a PHBHHx film produced with compression molding.; Figure S5: FTIR spectra of different ZnO nanoparticles. Spectra are not baseline corrected and normalized.; Figure S6: TGA measurements of different ZnO nanoparticles.; Table S6: Estimated hydroxyl concentration on the different ZnO NP types determined via TGA.; Figure S7: FTIR spectra in the wavenumber range 1150–1000 cm^−1^ for (i) PHBHHx and 5 wt.% PHBHHx/ZnO nanocomposites: (ii) ZnO(R), (iii) ZnO(R-Si), (iv) ZnO(R-Si-e), (v) ZnO(S-1), and (vi) ZnO(S-2).; Table S8: Qualitative crystallinity fraction (XC, FTIR) obtained from the C=O region and crystallinity indices I1227/I1453 and I1379/I1180 from the C-O-C region for PHBHHx/ZnO nanocomposites with ZnO concentrations of 1, 3, and 5 wt.%.; Figure S9: WAXD patterns for PHBHHx and ZnO nanocomposites with a ZnO concentration of 5 wt.%. (a) Characteristic peaks for PHBHHx and ZnO (2θ = 10–60°). (b) Zoom of the PHBHHx peaks (2θ = 10–30°). WAXD patterns are baseline corrected.; Table S9: WAXD calculated crystallinity (XC, XRD) for PHBHHx and their ZnO nanocomposites in different concentrations.; Table S10: TGA results of PHBHHx/ZnO nanocomposites showing the extrapolated onset degradation temperature (To), the temperature at 5% weight loss (T5%), the temperature at 50% weight loss (T50%) and the maximum peak degradation temperature (Tp).; Figure S11. UV–VIS spectra for 1 wt.% (a) and 3 wt.% (b) PHBHHx/ZnO nanocomposite films.; Figure S12: Exemplary images of PHBHHx/ZnO films: (a) 1 wt.% ZnO(S-2) visual appearance with (b) contact transparency showing the translucent nature and size of the film (10 × 10 cm^2^), and contact transparency of (c1) PHBHHx—opacity ~13%, (c2) 3 wt.% ZnO(R-Si)—opacity ~31% and (c3) 5 wt.% ZnO(S-2)—opacity ~58%.; Table S13: Minimum inhibitory concentrations (MIC) of the different ZnO NP types for S. aureus and E. coli. Standard deviation is shown in brackets.
