# Supplementary material for: Fabrication of Poly(3-hydroxybutyrate-co-3-hydroxyhexanoate)/ZnO Nanocomposite Films for Active Packaging Applications: Impact of ZnO Type on Structure–Property Dynamics
Source: Polymers (Basel). 2024 Jun 29;16(13):1861. doi: 10.3390/polym16131861 (PMC11243840; doi:10.3390/polym16131861)
Supplement: Supplementary file 1 [file polymers-16-01861-s001.zip › polymers-3060406-supplementary.pdf]

Fabrication of Poly(3-hydroxybutyrate-co-3-hydroxyhexanoate)/ZnO Nanocomposite Films for Active Packaging Applications: Impact of ZnO type on Structure-Property Dynamics

Chris Vanheusden[1], Pieter Samyn[2], Thijs Vackier[3], Hans Steenackers[3], Jan D'Haen[4], Roos Peeters[1] and Mieke Buntinx[1,*]

[1] Materials and Packaging Research & Services, Institute for Materials Research (IMO-IMOMEC), Hasselt University, Wetenschapspark 27, Diepenbeek 3590, Belgium; chris.vanheusden@uhasselt.be ; roos.peeters@uhasselt.be
[2] Department Circular Economy and Renewable Materials, SIRRIS, , Gaston Geenslaan 8, 3001 Leuven, Belgium; pieter.samyn@sirris.be
[3] Department of Microbial and Molecular Systems, Centre of Microbial and Plant Genetics (CMPG), KU Leuven, Leuven, Belgium; thijs.vackier@kuleuven.be ; hans.steenackers@kuleuven.be
[4] Analytical & Microscopical Services, Institute for Materials Research (IMO-IMOMEC), Hasselt University, Wetenschapspark 1, Diepenbeek 3590, Belgium. jan.dhaen@uhasselt.be
* Correspondence: mieke.buntinx@uhasselt.be

Supplementary Materials S1. Calculation of crystallinity via XRD

The degree of crystallinity ($X_{C,XRD}$) was calculated using the following equation (E_{S1a}):

$$X_{C,XRD}(\%) = \frac{S_C}{S_C + S_A} \times 100 \qquad (E_{S1a})$$

with S_C and S_A the integrated area under the crystalline peaks and amorphous halo, respectively. The "crystal" sizes (D_{hkl}) at the main peaks (020) and (110) were calculated following the Scherrer equation (E_{S1b}):

$$D_{hkl} = \frac{k\lambda}{\beta \cos(\theta)} \qquad (E_{S1b})$$

with D_{hkl} the "crystal" size in normal direction to the (hkl) lattice plane, k the Scherrer's constant (here with k = 0.9), λ the Cu radiation wavelength (1.54 Å) and β the full width at half maximum of the peak in radians. XRD spectra were first baseline corrected before analysis. Origin Pro® software (Version V9.7.0.188) was used for data processing.

Supplementary Materials S2. TEM of ZnO NP and size distribution analysis

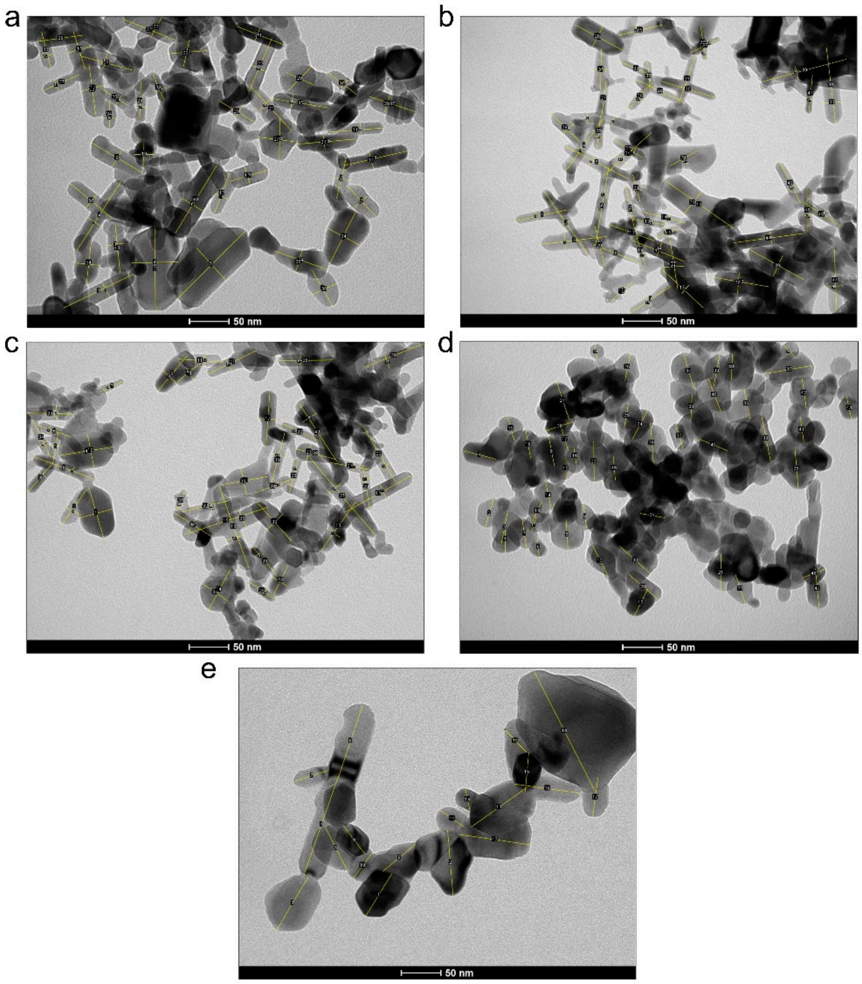

Figure S2. Exemplary ImageJ analysis of TEM images from ZnO NPs: (a) ZnO(R), (b) ZnO(R-Si), (c) ZnO(R-Si-e), (d) ZnO(S-1) and (e) ZnO(S-2). The yellow lines indicate measurements of ZnO NP rod lengths or diameters, depending on ZnO morphology (rod vs. spherical morphology).

Supplementary Materials S3. SEM PHBHHx/ZnO size distribution analysis

The size distribution of ZnO NPs in the PHBHHx matrix at 5 wt.% loading is shown in Figure S3.

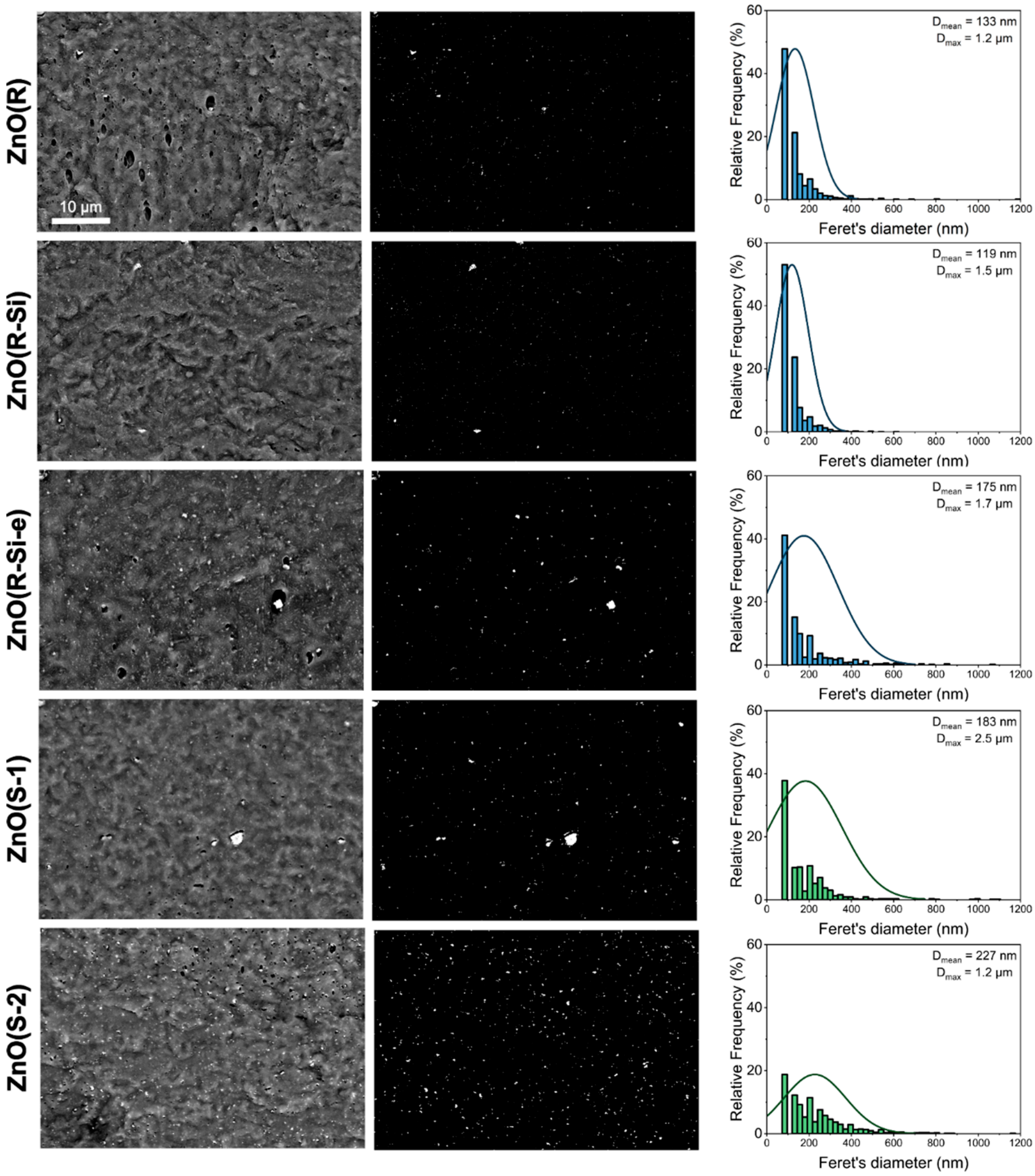

Figure S3. SEM size distribution analysis for PHBHHx/ZnO nanocomposites with different ZnO types at a concentration of 5 wt.%

Raw SEM images were transformed via Fiji ImageJ software (Version 1.53t) via threshold to black and white images, with the black background representing PHBHHx polymer and the white dots ZnO NPs. Particle analysis was performed by using Fiji ImageJ software (Version 1.53t) and the Feret's diameter for every countable ZnO NP was measured. It is notable that the average NP size is only an estimation because this method is limited to the black/white threshold between the polymer and the NP.

Supplementary Materials S4. FTIR spectrum of PHBHHx

The FTIR spectrum of PHBHHx (Figure S4) is clearly characterized by an ester function (C=O) at 1720 cm^{-1} [59]. The peaks at 1130 cm^{-1}, 1180 cm^{-1}, 1227 cm^{-1}, 1379 cm^{-1}, and 1453 cm^{-1} arise from C-O-C symmetric stretching, C-O-C asymmetric stretching, conformational band of helical chains, CH_3 symmetric wagging, and CH_3 asymmetric bending respectively [59].

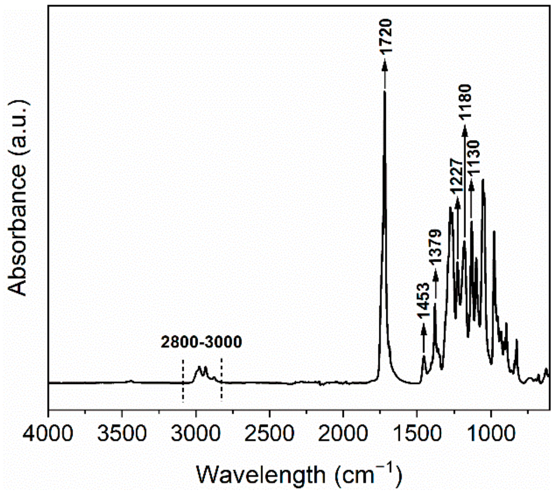

Figure S4. Detailed FTIR spectrum of a PHBHHx film produced with compression molding.

Supplementary Materials S5. FTIR spectra of ZnO nanoparticles

The FTIR spectra of different ZnO NPs used in this study are shown in Figure S. The FTIR spectrum of ZnO NPs is generally characterized by an absorbance band around 450-600 cm^{-1} from Zn-O stretching and an absorbance band around 880 cm^{-1} assigned to Zn-OH stretching. Different types of hydroxyl (-OH) groups can be located on the surface of ZnO NPs. A first type is found at the absorbance band around 3000-3650 cm^{-1} from reversible dissociative adsorption of hydrogen on both Zn and O sites [62]. This band is apparent for all ZnO NP types but with rather low intensity, indicating a relatively low amount of -OH groups on the surface of all tested ZnO NPs. Hydroxyl groups can also be located on the surfaces as chemisorbed water [60-62], and apparent in the FTIR spectra as a rather small band around 1640 cm^{-1} for the unmodified ZnO(R) and ZnO(S-1). The peak at 1740 cm^{-1} can be assigned to stretching vibrations of C=O bonds. The three intensities at wavenumbers of 2850-3000 cm^{-1} are assigned to C-H stretching, possibly from acetate ions [62].

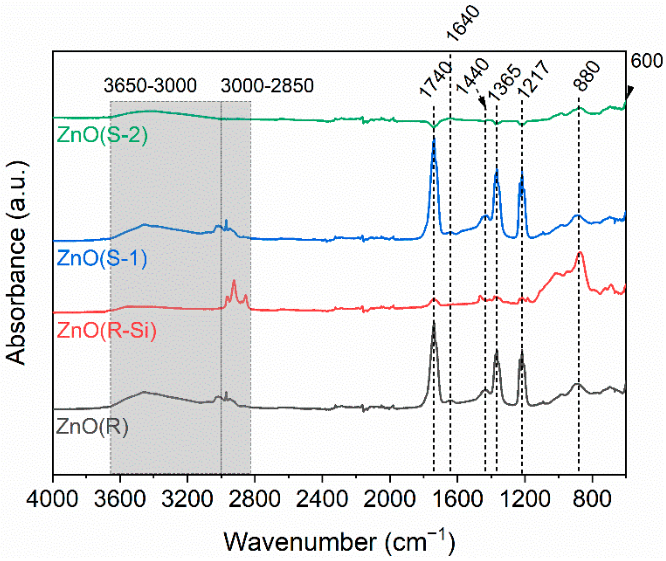

Figure S5. FTIR spectra of different ZnO nanoparticles. Spectra are not baseline corrected and normalized.

Supplementary Materials S6. TGA of ZnO nanoparticles

The amount of hydroxyl groups on the surface of ZnO NPs can be determined via TGA measurements (Figure S6). The ZnO powders were not dried prior to analysis. The weight loss remains below 2% for all ZnO types, indicating a low amount of physiosorbed and chemisorbed water on the surface of ZnO. The ZnO(R-Si) and ZnO(R-Si-e) types show higher weight losses due to surface modification with silane. The amount of chemisorbed water (and hydroxyl concentration) can be assessed from the TGA curves (Table S6) in the temperature interval of 180°C-500°C and can be calculated with the following equation E_{S6} [48] (not determined for ZnO(R-Si) and ZnO(R-Si-e) to avoid overlap with weight loss due to silane):

$$[OH] = \left(\frac{2}{M_{H_2O}}\right) \times \left(\frac{weight\ loss\ (\%)}{100 - weight\ loss\ (\%)}\right) \qquad (E_{S6})$$

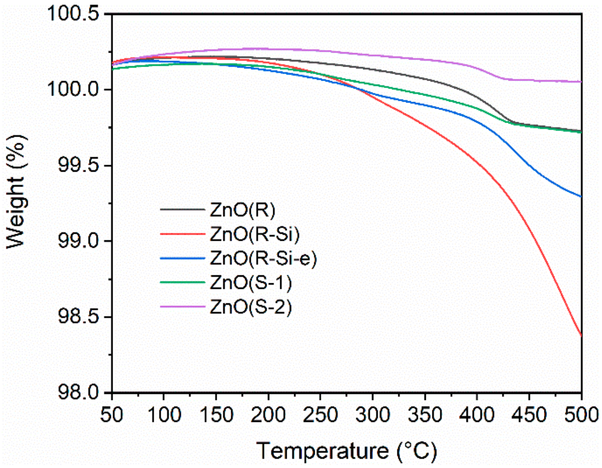

Figure S6. TGA measurements of different ZnO nanoparticles.

Table S6. Estimated hydroxyl concentration on the different ZnO NP types determined via TGA.

ZnO type	Weight loss TGA* (wt.%)	[OH] (mmol/g)
ZnO(R)	0.486	0.542
ZnO(S-1)	0.443	0.494
ZnO(S-2)	0.218	0.243

*Weight loss in the temperature interval of 180-500°C.

Supplementary Materials S7. FTIR spectra 5 wt.% PHBHHx/ZnO (1150-1000 cm^{-1})

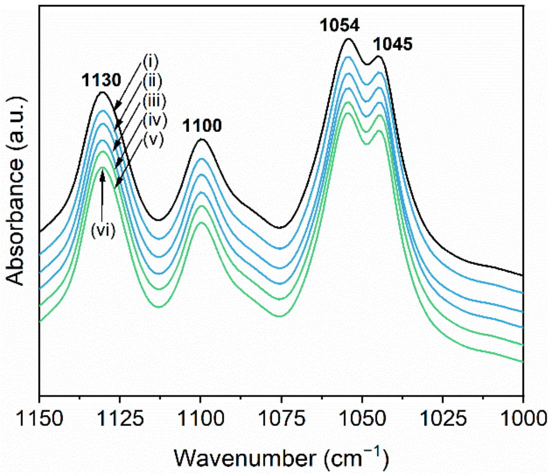

Figure S7. FTIR spectra in the wavenumber range 1150-1000 cm^{-1} for (i) PHBHHx and 5 wt.% PHBHHx/ZnO nanocomposites: (ii) ZnO(R), (iii) ZnO(R-Si), (iv) ZnO(R-Si-e), (v) ZnO(S-1) and (vi) ZnO(S-2).

Supplementary Materials S8. Determination of crystallinity in FTIR

Bands at lower wavenumbers (1720 cm^{-1}) are assigned to the crystalline phase, where O atoms of the C=O group are closer located to H atoms to form H-bond interactions (reduction of the C=O order) [65]. The wider bands at higher wavenumbers (e.g. 1741 cm^{-1}) are assigned to the amorphous phase, where H-bonding interactions are reduced due to absence of an ordered structure (increase of the C=O order) [65]. In this way, the C=O region of semicrystalline PHBHHx is a superimposition of the crystalline and amorphous spectral components and their ratio can be used for a qualitative crystallinity fraction ($X_{C,FTIR}$) of the nanocomposites, and was determined in the ester (C=O) spectral region by a multiple Lorentz peak fit performed with Origin Pro® software (Version V9.7.0.188). The crystallinity fraction $X_{C,FTIR}$ was determined using following equation E_{S8}:

$$X_{C,FTIR}(\%) = \frac{A_C}{A_C + A_A} \times 100 \qquad (E_{S8})$$

with A_C and A_A the integrated area of the crystalline and amorphous fraction respectively. The difference in absorptivity between the crystalline and amorphous phase was not taken into account [65].

Another qualitative assessment of the crystallinity was assessed in the C-O-C spectral region and the crystallinity index (CI) was determined as the ratio of I_{1227}/I_{1453} and I_{1379}/I_{1180}.

Table S8. Qualitative crystallinity fraction ($X_{C,FTIR}$) obtained from the C=O region and crystallinity indices I_{1227}/I_{1453} and I_{1379}/I_{1180} from the C-O-C region for PHBHHx/ZnO nanocomposites with ZnO concentrations of 1, 3 and 5 wt.%.

Sample	C_{ZnO} (wt.%)	$X_{C,FTIR}$	I_{1227}/I_{1453}	I_{1379}/I_{1180}
PHBHHx	0	77.1	0.56	4.48
ZnO(R)	1	77.1	0.62	4.71
	3	79.6	0.60	4.57
	5	80.1	0.59	4.47
ZnO(R-Si)	1	79.6	0.61	4.59
	3	80.1	0.60	4.60
	5	82.9	0.61	4.72
ZnO(R-Si-e)	1	79.5	0.60	4.53
	3	76.6	0.58	4.79
	5	83.3	0.60	4.57
ZnO(S-1)	1	85.5	0.59	4.43
	3	80.2	0.61	4.74
	5	78.1	0.58	4.75
ZnO(S-2)	1	78.4	0.57	4.82
	3	79.3	0.58	4.82
	5	79.7	0.58	4.77

Supplementary Materials S9. WAXD spectra of PHBHHx/ZnO and crystallinity

The WAXD spectra of PHBHHx and PHBHHx/ZnO films at a ZnO loading of 5 wt.% are shown in Figure S9. The spectra show similar patterns compared to the PHB homopolymer, indicating the formation of orthorhombic PHB unit cells in these film samples. The XRD pattern of PHBHHx is mainly characterized by reflections: (020) at 2θ = 13.6°, (110) at 2θ = 17°, (111) at 2θ = 22.5°, (031) at 2θ = 25.7° and (040) at 2θ = 27.2° [59]. The two most intense scattering peaks (020) and (110) indicate the orthorhombic unit cell of PHB. The reflections of the ZnO nanocomposites (both 1 and 5 wt.%) have not shifted and peak intensities are comparable, indicative of a similar crystallinity as in PHBHHx and confirming the presented DSC and FTIR data. The crystallinity values as calculated from the crystalline peaks in the XRD pattern remain fairly constant for all samples (~60 to65 %). In addition, the "crystal" sizes as calculated with the Scherrer equation remain also similar, estimated to be around 23 ± 1 nm and 14 ± 1 nm, for (020) and (010), respectively and confirming the presented DSC data.

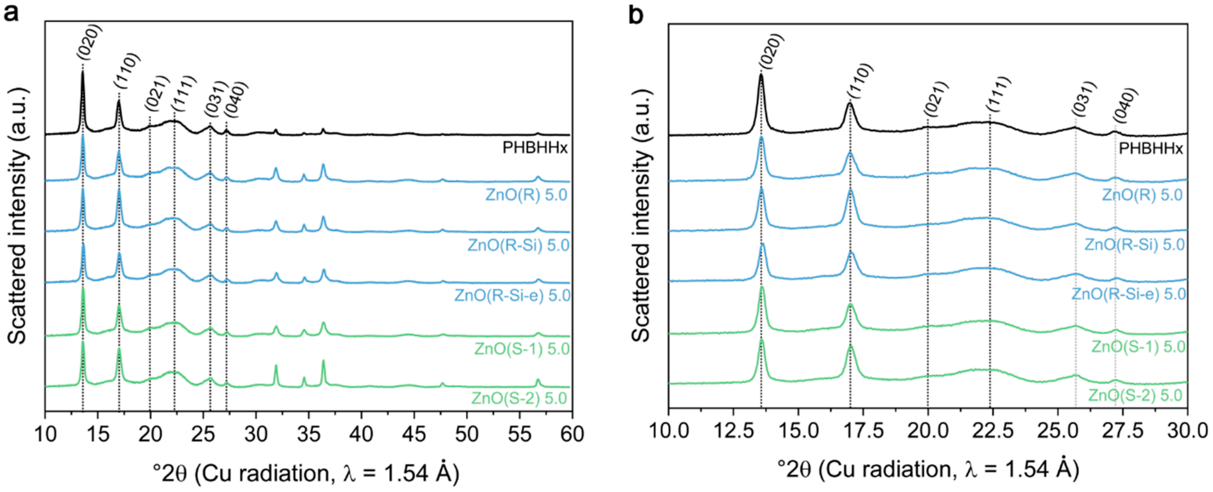

Figure S9. WAXD patterns for PHBHHx and ZnO nanocomposites with a ZnO concentration of 5 wt.%: (a) characteristic peaks for PHBHHx and ZnO (2θ = 10-60°) and (b) zoom of the PHBHHx peaks (2θ = 10-30°). WAXD patterns are baseline corrected.

Table S9. WAXD calculated crystallinity ($X_{C,XRD}$) for PHBHHx and their ZnO nanocomposites in different concentrations.

Sample	C_{ZnO} (wt.%)	$X_{C.XRD}$ (%)
PHBHHx	0	61.8
ZnO(R)	5	59.1
ZnO(R-Si)	1	64.0
	5	60.1
ZnO(R-Si-e)	5	61.2
ZnO(S-1)	5	63.7
ZnO(S-2)	1	65.4
	5	63.5

Supplementary Materials S10. TGA characteristic values of PHBHHX/ZnO nanocomposites

Table S10. TGA results of PHBHHx/ZnO nanocomposites showing the extrapolated onset degradation temperature (T_o), the temperature at 5% weight loss ($T_{5\%}$), the temperature at 50% weight loss ($T_{50\%}$), the maximum peak degradation temperature (T_p) and residue (R).

Sample	C_{ZnO} (wt.%)	T_o (°C)	$T_{5\%}$ (°C)	$T_{50\%}$ (°C)	T_p (°C)	R(%)
PHBHHx	0	292	286	304	308	0.4
ZnO(R)	1	281	273	293	297	1.1
	3	275	270	289	290	3.0
	5	276	273	292	290	4.7
ZnO(R-Si)	1	282	275	293	296	1.2
	3	277	272	290	289	2.8
	5	278	275	293	291	4.9
ZnO(R-Si-e)	1	281	273	293	297	1.2
	3	275	271	289	288	2.9
	5	277	274	292	290	4.8
ZnO(S-1)	1	281	273	293	297	1.2
	3	275	270	289	289	3.6
	5	276	274	292	290	4.7
ZnO(S-2)	1	280	273	292	297	1.2
	3	276	271	289	290	2.8
	5	274	272	290	286	5.0

Supplementary Materials S11. UV/VIS spectra of PHBHHx/ZnO films

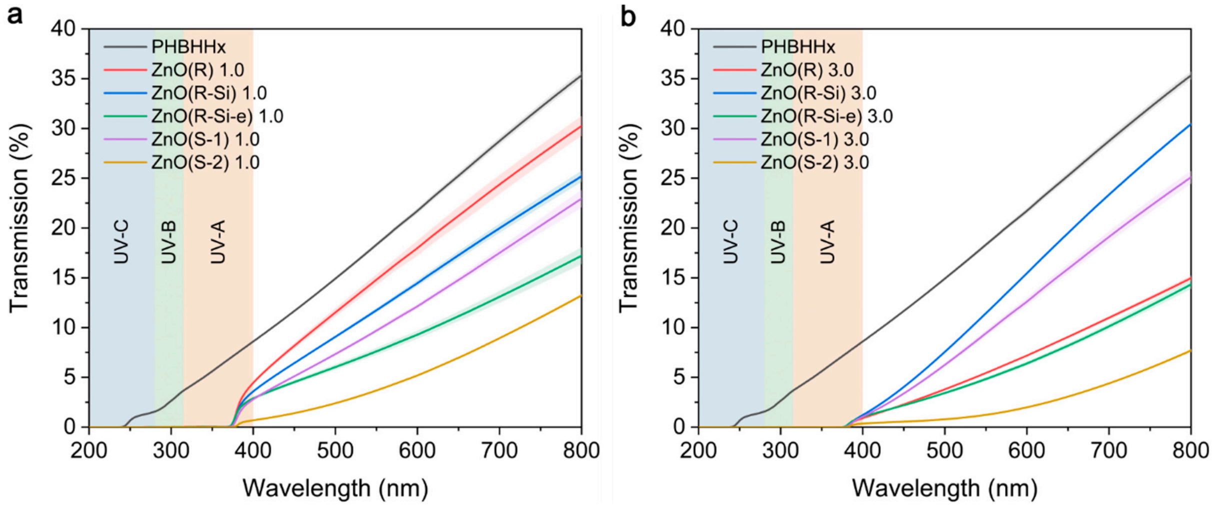

Figure S11. UV-VIS spectra for 1 wt.% (a) and 3 wt.% (b) PHBHHx/ZnO nanocomposite films. The curves are averaged (n = 3) and the shaded colors represent the SD.

Supplementary Materials S12. Optical characteristics of PHBHHx/ZnO films

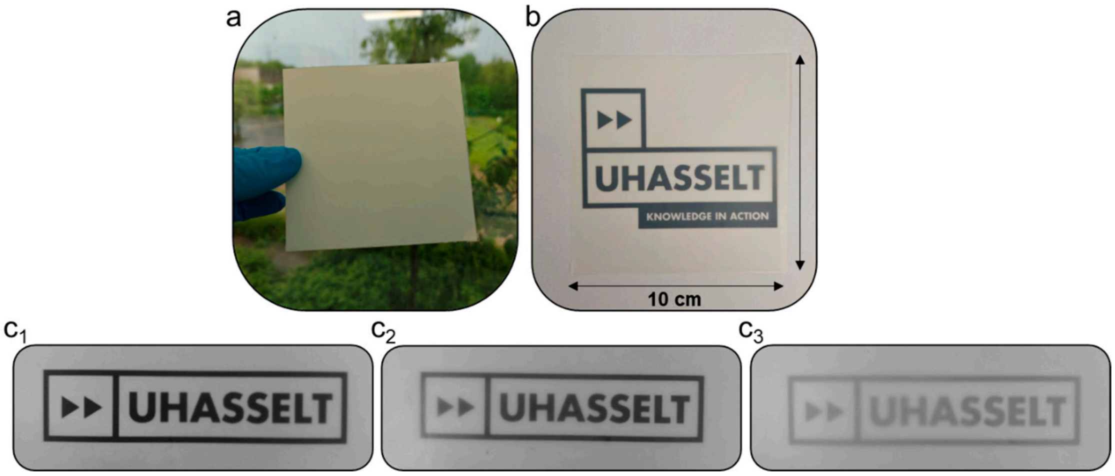

Figure S12. Exemplary images of PHBHHx/ZnO films: (a) 1 wt.% ZnO(S-2) visual appearance with (b) contact transparency showing the translucent nature and size of the film (10 x 10 cm^2), and contact transparency of (c_1) PHBHHx – opacity ~13%, (c_2) 3 wt.% ZnO(R-Si) – opacity ~ 31% and (c_3) 5 wt.% ZnO(S-2) – opacity ~ 58%.

Supplementary Materials S13. Minimum inhibitory concentrations of ZnO NPs

Table S13. Minimum inhibitory concentrations (MIC) of the different ZnO NP types for *S. aureus* and *E. coli*. Standard deviation is shown in brackets.

ZnO type	MIC (µg/ml)	
	S. aureus	*E. coli*
ZnO(R)	500 (0)	1000 (0)
ZnO(R-Si)	1333.3 (577)	>2000
ZnO(R-Si-e)	>2000	>2000
ZnO(S-1)	2000 (0)	>2000
ZnO(S-2)	416.7 (144)	2000 (1732)

References

59. Díez-Pascual, A.M. Effect of Graphene Oxide on the Properties of Poly(3-Hydroxybutyrate-Co-3-Hydroxyhexanoate). *Polymers* **2021**, *13*, 2233, doi:10.3390/polym13142233.
62. Pourrahimi, A.M.; Liu, D.; Ström, V.; Hedenqvist, M.S.; Olsson, R.T.; Gedde, U.W. Heat Treatment of ZnO Nanoparticles: New Methods to Achieve High-Purity Nanoparticles for High-Voltage Applications. *J. Mater. Chem. A* **2015**, *3*, 17190–17200, doi:10.1039/C5TA03120F.
60. Ramimoghadam, D.; Bin Hussein, M.Z.; Taufiq-Yap, Y.H. Hydrothermal Synthesis of Zinc Oxide Nanoparticles Using Rice as Soft Biotemplate. *Chemistry Central Journal* **2013**, *7*, 136, doi:10.1186/1752-153X-7-136.
61. Lee, P.; Saion, E.; Al-Hada, N.; Soltani, N. A Simple Up-Scalable Thermal Treatment Method for Synthesis of ZnO Nanoparticles. *Metals* **2015**, *5*, 2383–2392, doi:10.3390/met5042383.
48. Bressy, C.; Ngo, V.G.; Ziarelli, F.; Margaillan, A. New Insights into the Adsorption of 3-(Trimethoxysilyl)Propylmethacrylate on Hydroxylated ZnO Nanopowders. *Langmuir* **2012**, *28*, 3290–3297, doi:10.1021/la204544c.
65. Kansiz, M.; Domínguez-Vidal, A.; McNaughton, D.; Lendl, B. Fourier-Transform Infrared (FTIR) Spectroscopy for Monitoring and Determining the Degree of Crystallisation of Polyhydroxyalkanoates (PHAs). *Anal Bioanal Chem* **2007**, *388*, 1207–1213, doi:10.1007/s00216-007-1337-5.
